# Unfounded authority, underpowered studies, and non-transparent reporting perpetuate the Mozart effect myth: a multiverse meta-analysis

**DOI:** 10.1038/s41598-023-30206-w

**Published:** 2023-03-06

**Authors:** Sandra Oberleiter, Jakob Pietschnig

**Affiliations:** Department of Developmental and Educational Psychology, Faculty of Psychology, University of Vienna, 1010 Vienna, Austria

**Keywords:** Psychology, Human behaviour

## Abstract

In recent years, an ostensible Mozart effect, suggesting beneficial influences of listening to the sonata KV448 on epilepsy, has been extensively covered in popular media outlets. However, the evidential value of such a potential effect seems unclear. Here, we present the first formal meta-analysis on this topic, based on *k* = 8 studies (*N* = 207). Further published studies that met our inclusion criteria had to be omitted due to insufficient reporting and author non-responsiveness on data requests. In three independent analyses, we observed non-significant trivial-to-small summary effects for listening to Mozart KV448 or other musical stimuli on epilepsy or other medical conditions (*g* range: 0.09–0.43). Bias and sensitivity analyses suggested that these effects were likely inflated and non-trivial effects were driven by isolated leverage points. Multiverse analyses conformed to these results, showing inconsistent evidential patterns. Low primary study power and consequently lacking evidential value indicates that there is only little reason to suspect a specific Mozart effect. In all, listening to music, let alone a specific kind of sonata, does not appear to have any beneficial effect on epilepsy. Unfounded authority, underpowered studies, and non-transparent reporting appear to be the main drivers of the Mozart effect myth.

## Introduction

Over the past three decades, the Mozart effect has generated a lot of attention both in the scientific community and in popular media. The topic was introduced in the context of spatial task performance^1^, which supposedly improved after subjects were exposed to the first movement “allegro con spirito” of Mozart’s sonata KV448. This phenomenon was received with considerable skepticism in the scientific community and ultimately demonstrated to be a consequence of low study power and bias-related measurement artifacts^2^. However, claims about long-lasting intelligence-boosting effects, especially in children, have popularized the Mozart effect in the public with a small industry piggy-backing on the ostensible phenomenon by selling tailored selections of allegedly cognitive performance-enhancing classical music^3^. To this very day, the public interest in the Mozart effect and the supposedly beneficial effect of Mozart’s music on intelligence remains unabated.

Perhaps as a consequence of this public interest, effects of listening to Mozart’s music have also been investigated in regard to many other outcomes besides intelligence, with potentially symptom-alleviating effects in epilepsy being among the most frequently cited ones that have also generated considerable attention in public outlets^4^. Originally introduced in the late 1990s with some results suggesting that listening to KV448 leads to an acute decrease in both ictal and interictal epileptiform activity^5^, at least two studies have so far reported successful replications of this effect^6,7^.

A Mozart effect for epilepsy would be desirable because antiepileptic drugs often cause severe side effects and may have a negative impact on organ functions, fertility, or blood counts of patients^8^. For 30% of those affected by epilepsy, drug therapies are ineffective^9^. Some authors have suggested that KV448 may be used to supplement or replace drug treatment when medication or surgery were ineffective or would not be accepted^10^. In some studies, patients listening to KV448 were reported to have experienced fewer epileptic seizures and epileptic discharges compared to patients who waited in silence or listened to other music^11,12^, whilst *Haydn's Symphony No. 94* was even reported to act pro-epileptic^13^. Other studies contrast these findings, indicating no specific beneficial effect of KV448 on epilepsy^14^.

To date, two narrative reviews are available about KV448 effects on epilepsy: In the first one, results of eight studies (seven of which are first-authored by the same person) are summarized, suggesting positive effects of listening to KV448 on epilepsy^10^. The second review used a vote counting approach based on nine studies and arrived at the same conclusion^9^. However, no formal meta-analytic effect syntheses are currently available which means that an evaluation of effect strength, meaningfulness, or potentially confounding bias is unavailable, thus raising concerns about the validity of these past conclusions.

Consequently, here we present a systematic review and meta-analysis of KV448 effects on epilepsy and related medical conditions. Moreover, we provide evidence for potential influences of (i) dissemination biases, (ii) the adequacy of the evidential value, as well as (iii) different ways about which data were analyzed and how this has been done by means of multiverse analyses.

## Methods

The present study was preregistered prior to accessing the data. The preregistration protocol and any deviations from the preregistration are documented at the Open Science Framework (OSF; https://osf.io/t328m and https://osf.io/ry8m5). A PRISMA (Preferred Reporting Items for Systematic Reviews and Meta-Analyses) checklist can be obtained from Table S1 (https://osf.io/72mgx). Primary study quality was assessed with the Newcastle–Ottawa Scale^15^ (Table S4; https://osf.io/u3g64).

### Research question

In accordance with the PICO (Population, Intervention, Comparison, Outcome) statement, our literature search and critical assessment was based on the following research question: “In patients with epilepsy or other medically relevant conditions (P), does the exposure to the first movement “allegro con spirito” of Mozart’s sonata KV448 (I), compared with patients exposed to (i) another musical stimulus, (ii) a non-musical stimulus, or (iii) silence (C), improve their symptomatology (O)?”.

### Literature search

**Figure 1:**
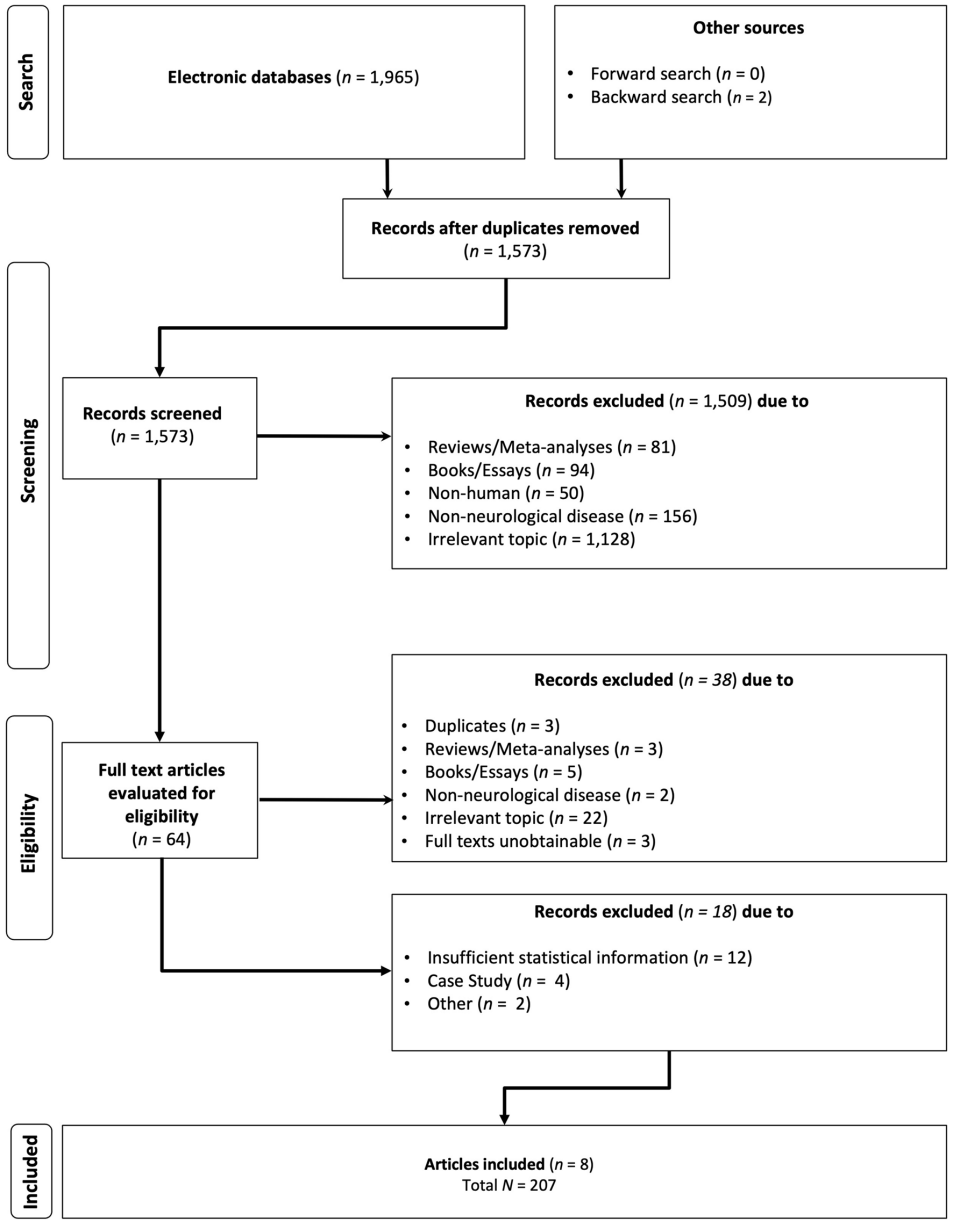
Flow-chart of the study identification and selection process, following Preferred Reporting Items for Systematic Reviews and Meta-Analyses (PRISMA) guidelines.

We searched six databases for published studies (Google Scholar, PubMed, Scopus, ISI Web of Science, PsycInfo, PubPsych) and the Open Access Theses and Dissertation database to obtain grey literature (https://oatd.org). First, we used the following search string to identify relevant literature: (“mozart effect” AND epil*) OR (“mozart effect” AND brain) OR (“mozart effect” AND disease). Second, we screened the reference lists of studies that were eligible for inclusion in our synthesis for further potentially relevant hits. Finally, we conducted a cited reference search for the initial study that had been published on the Mozart effect^1^ as well as the so far largest meta-analysis on this topic^2^. Non-English or -German titles, abstracts, and fulltexts were translated with DeepL (https://deepl.com/translator). Titles and abstracts of 1573 potentially relevant articles were screened and subsequently fulltexts of 64 studies were obtained (flowchart in Fig. 1; references of excluded records according to exclusion criteria are provided at https://osf.io/vugm7). Literature search and screening were originally conducted from June to July 2022 and updated in October 2022.

### Inclusion and exclusion criteria

To be eligible for inclusion in the present meta-analysis, studies had to meet three inclusion criteria. First, they had to assess the effects of listening to Mozart’s sonata KV448, another musical stimulus, a non-musical stimulus (e.g., listening to a short story), or silence on a medically relevant condition. Second, studies had to provide an appropriate measure for the symptoms of the respective medical condition, such as the number of epileptiform seizures experienced or interictal epileptiform discharges (IED) in case of epilepsy. Third, effect sizes or sufficient statistical information to calculate them needed to be available.

Studies were excluded from analysis if they (i) did not include a control condition in their design, (ii) did not provide any measurement of patient symptom changes, or (iii) did not report (or study authors did not provide upon request) sufficient statistical information to calculate effect sizes.

### Coding

The coding of the studies was conducted twice independently by the same experienced researcher [S.O.]. Coding inconsistencies were resolved through discussion with an independent coder [J.P.]. The following information were coded for individual studies: (i) study characteristics (publication status: published vs. unpublished; publication year; manuscript type: journal article vs. thesis; peer-reviewed: yes vs. no; funding: not reported vs. yes vs. no;), (ii) country of data collection, (iii) sample descriptors (sample size, mean age, sample type, percentage of men within sample), (iv) epilepsy or physical disease measurement (type of disease: epilepsy vs. other; seizure type: generalized and focal vs. other vs. mixed; type of control stimulus: other classical vs. non classical / scrambled; duration of exposure), and (vi) statistical parameters (pre- and posttest means, standard deviations, effect sizes, *p*-values, reliabilities of dependent variables). In case of missing information, the primary studies’ corresponding authors were contacted and reminders were sent after two and four weeks if no response had been received. If data were unavailable or the corresponding authors did not reply, the respective study was excluded from analyses (coding file and study information available at https://osf.io/t5wyb).

### Data analysis

Prior to all analyses, Hedges *g*s were calculated for group differences^16^. Data were synthesized according to (i) the different stimuli the experimental groups were exposed to and (ii) the study design that was applied. Three independent meta-analyses were conducted: First, we meta-analyzed primary studies that compared effects of listening to KV448 versus silence in independent-groups pretest–posttest designs (henceforth: *independent MO-condition*; *k* = 3 study effects). Second, we once more meta-analyzed KV448 versus silence studies but synthesized studies that used one-group pretest–posttest designs only (henceforth: *dependent MO-condition*; *k* = 5 study effects). Third, we synthesized effects of listening to any other music versus no stimulus at all in one-group pretest–posttest designs (*OM-condition*; *k* = 6 study effects). The three outcomes of the independent MO-condition were based on studies investigating KV448 effects on either epilepsy, blood pressure of stroke patients, or other-reported premature infant pain. In both the dependent MO-condition and the OM-condition, all outcomes pertained to effects on epilepsy.

If there is indeed a salient specific Mozart effect, we should be able to observe a meaningful significant effect in both MO-conditions, but no effect in the OM-condition.

Effect sizes of the samples were weighted by study precision (i.e., assigning higher weights to more precise studies according to the inverse standard errors of effect sizes) and synthesized in random-effects models. Potential effects of leverage points were assessed by means of leave-one-out analyses.

We interpret between-studies heterogeneity according to well-established thresholds for *I*^2^ values (i.e., 25%, 50%, and 75% representing the lower thresholds of small, moderate, and large heterogeneity^17^) and prediction intervals^18^.

We conducted a series of subgroup analyses, to assess potential influences of categorical moderator variables (see Table 1). Potential influences of continuous moderators were examined by means of linear precision-weighted meta-regressions (for an overview, see Table 1; within-subgroup summary effect estimates and meta-regression effects are only provided if *k* > 1 and > 2, respectively).

**Table 1:** Moderator and specification-relevant variables for three independent meta-analyses.

Independent MO-condition (*k* = 3)	Dependent MO-condition (*k* = 6)	OM-condition (*k* = 5)
Categorical moderators		
-	Measurement method (seizure frequency vs. IED-EEG)	Measurement method (seizure frequency vs. IED-EEG)
-	Funding (not reported/no vs. yes)	Funding (not reported/no vs. yes)
-	Sample type (adults vs. mixed)	Sample type (adults vs. children)
-	Seizure type (generalized and focal vs. not reported/mixed)	Type of control music (other classical vs. non-classical/scrambled)
Continuous moderators		
Publication year	Publication year	Publication year
Age	Age	Age
Percentage of men in samples	Percentage of men in samples	Percentage of men in samples
Duration of exposure	Duration of exposure	Duration of exposure
Specifications—*which* factors		
Type of disease (epilepsy vs. other vs. either)	Seizure type (generalized/focal vs. other vs. mixed vs. either)	Seizure type (mixed vs. other vs. either)
Measurement method (other than IED-EEG vs. either)	Measurement method (IED-EEG vs. seizure frequency vs. either)	Measurement method (IED-EEG vs. other vs. either)
Exposure (more than once vs. once vs. either)	Sample type (adults vs. mixed vs. either)	Exposure (more than once vs. either)
Sample type (children vs. adults vs. either)	Funding (yes vs. not reported vs. either)	Sample type (children vs. adults vs. either)
Funding (not reported vs. yes vs. no vs. either)		Funding (yes vs. no vs. either)
		Type of control music (other classical vs. scrambled vs. either)
Specifications—*how* factors		
Effect metric (Hedges *g* vs. Cohen *d*)	Effect metric (Hedges *g* vs. Cohen *d*)	Effect metric (Hedges *g* vs. Cohen *d*)
Approach (HO vs. HS vs. FE)	Approach (HO vs. HS vs. FE)	Approach (HO vs. HS vs. FE)

Independent MO = independent-groups pretest–posttest designs examining KV448 versus silence^11,27,28^; Dependent MO = one-group pretest–posttest designs examining KV448 versus silence^7,12,25,26^, OM = one-group pretest–posttest designs examining other music versus silence^12,25,26,29^; positive signs indicate a beneficial effect of KV448 (MO) and other music (OM) compared to silence; *k* = number of effect sizes included in the respective condition; IED-EEG = interictal epileptic discharges measured with an electroencephalogram; scrambled = phase-scrambled version of KV448 representing a control piece with noise but no rhythmicity^25^; HO = Hedges and Olkin-typed random-effects estimation (REML); HS = Hunter-Schmidt effect estimation; FE = fixed-effect model.

To detect potential influences of confounding dissemination bias, we used different bias detection approaches (in all ten; see technical supplementary material available at https://osf.io/b8ury) following current recommendations from the literature^19^ to account for the different strengths and weaknesses of individual approaches. Only published studies were included in our publication bias analysis.

Because there are different (reasonable) ways of which studies to include and how to synthesize them in a meta-analysis, all of which may affect the results and interpretation of the outcomes^20^, we used multiverse analyses to account for potential outcome differences according to different specifications (see https://osf.io/nkv46/ for the R Code^20^).

We used specification curve analyses to assess the effect of different reasonable combinations of which data to analyze and how to do this (so-called *which* and *how factors*). In this approach, it is assumed that all specifications that are based on the combination of any levels of different conceptually plausible moderators may be assumed to be equally reasonable (in other words: all summary effects are equally likely to reflect reality most accurately; Table 1).

However, it could be argued that certain reasonable specifications may remain undetected by specification curve analyses, because not all reasonable specifications may be known. Therefore, we used combinatorial meta-analyses to assess potential systematic influences of any combination on our effect syntheses^21^. Typically, due to the astronomical number of possible (unreasonable) combinations in any meta-analysis, a sample of 100,000 ways to calculate summary effects is randomly drawn from the data and resulting effect patterns are visually inspected and distributional characteristics are interpreted. However, due to the low number of available data points in our analyses, we are able to provide here an exhaustive analysis of all possible combinations. By calculating summary effects for 2^k^–1 possible subsets of the available data, we obtained (i) 2^3^–1 = 7 combinations for the independent MO-condition, (ii) 2^6^–1 = 63 combinations for the dependent MO-condition, and (iii) 2^5^–1 = 31 combinations for the OM-condition.

All analyses were performed by means of the open-source software R^22^, the online app MetaShine^23^, and the *p*-curve app^24^.

## Results

### Final sample

We identified 26 studies that conformed to our inclusion criteria. Six studies provided sufficient statistical information to calculate a summary effect size^7,11,12,25–27^. From the remaining 20 studies we had to exclude four because they represented single case reports, whose data cannot be formally meta-analyzed (see Table S2 available at https://osf.io/nsu8y). Another study was excluded because we were unable to locate any contact information for any of the authors^5^. We contacted all corresponding authors (*k* = 7) of the remaining 15 studies: Two authors provided sufficient summary data upon request for three studies (one of which had to be excluded due to being the only study in our entire analysis that compared KV448 to other music^14^) in personal communications^28,29^. Two corresponding authors communicated to us that the data from their published studies were no longer accessible for them. Additionally, another author reported that this was also the case for six independent studies that had been published by him and his team. Four corresponding authors did not respond at all (for an overview, see Table S2).

Consequently, we could formally meta-analyze data of *k* = 8 (totaling *N* = 207 participants) studies, which assessed the Mozart effect and its relation to either epilepsy (*k* = 6), stroke (*k* = 1), or other-reported premature infant pain (*k* = 1). Studies observed patients’ seizure frequency (*k* = 3), evaluated interictal epileptic discharges by means of an electroencephalogram (IED-EEG; *k* = 3), or used other methods to measure changes in the respective symptomatology (*k* = 2). Study characteristics are detailed in Table 2 (we provide all data at https://osf.io/t5wyb).

**Table 2:** Study characteristics of included studies.

Reference	*N*	Type	Medical condition	Measure	Condition	Study design	Journal	ES (*SE*)	Data availability
Bergomi et al.^28^	70	Children	Other-reported pain in premature infants	Premature infant pain profile (PIPP)	Independent MO	Randomized controlled	Research and Theory for Nursing Practice	*g* = 1.655 (0.27)^b^	Summary data provided upon request
Coppola et al.^29^	11	Children	Epilepsy	Observation -seizure frequency	OM	One-group pretest–posttest	Epilepsy & Behavior	*g* = 0.743 (0.26)	Summary data provided upon request
D’Alessandro et al.^7^	12	Mixed	Epilepsy	Observation -seizure frequency	Dependent MO	Mirror-design (no washout)^a^	Psychiatria Danubina	*g*1 = 0.083 (0.34)^c^ *g*2 = 0.197 (0.35)	Summary data available in paper
Grylls et al.^26^	45	Children	Epilepsy	EEG—interictal epileptiform discharges	Dependent MO; OM	One-group counterbalanced (no washout)	Seizure	*g* (MO-NM) = 0.045 (0.15) *g* (OM-NM) = − 0.03 (0.14)^c^	Summary data available in paper
Paprad et al.^11^	26	Children	Epilepsy	EEG—interictal epileptiform discharges	Independent MO	Randomized controlled	Epilepsy & Behavior	*g* = 0.096 (0.38)^a^	Summary data available in paper
Rafiee et al.^25^	11	Adults	Epilepsy	Observation -seizure frequency	Dependent MO; OM	Mirror-design (no washout)	Epilepsia Open	*g*1(MO-NM) = 1.04 (0.48) *g*2(MO-NM) = 0.06 (0.34)^c^ *g*1(OM-NM) = 0.36 (0.38)^c^ *g*2(OM-NM) = − 0.13 (0.35)	Primary data available in paper
Stillova et al.^12^	18	Adults	Epilepsy	EEG—interictal epileptiform discharges	Dependent MO; OM	One-group counterbalanced (no washout)	European Journal of Neurology	*g* (MO-NM) = 0.30 (0.23)^b^ *g* (OM-NM) = − 0.21 (0.23)^b, c^	Summary data available in paper
Vibrasiute^27^	14	Adults	Stroke	Systolic blood pressure	Independent MO	Randomized controlled	Unpublished (bachelor’s thesis)	*g* = − 0.610 (0.52)	Primary data available in thesis

*N* = sample size; ES = Effect size; *SE* = standard error; Independent MO = independent-groups pretest–posttest designs examining KV448 versus silence; Dependent MO = one-group pretest–posttest designs examining KV448 versus silence, OM = one-group pretest–posttest designs examining other music versus silence; positive signs indicate a beneficial effect of KV448 (MO) or other music (OM) compared to silence; ^a^ = study was conceptualized as an RCT, but because no washout period was used, the study was assumed to represent a mirror design in our analyses; ^b^ = mean and standard deviation for calculating effect sizes are based on median and interquartile ranges according to the approach of Shi et al. (2020) and Luo et al. (2018); ^c^ = outcomes may be overestimates due to carry-over effects.

Of the includable studies, *k* = 3 used two-group randomized controlled designs (RCT). Only one study investigated RCT-based effects on epilepsy, whilst the other two remaining RCT’s investigated effects on other medically relevant conditions. Another four studies used two-group pre-post mirror- (*k* = 2) or one-group pre-post counterbalanced designs (*k* = 2) but did not control for potential carry-over effects by means of washout periods (i.e., between treatment and control condition, there was no pause that might have ensured a removal of potentially remaining effects of KV448; carry-over effects may invalidate conclusions of the respective studies). Another study used a one-group pretest–posttest design.

### Main analyses

**Figure 2:**
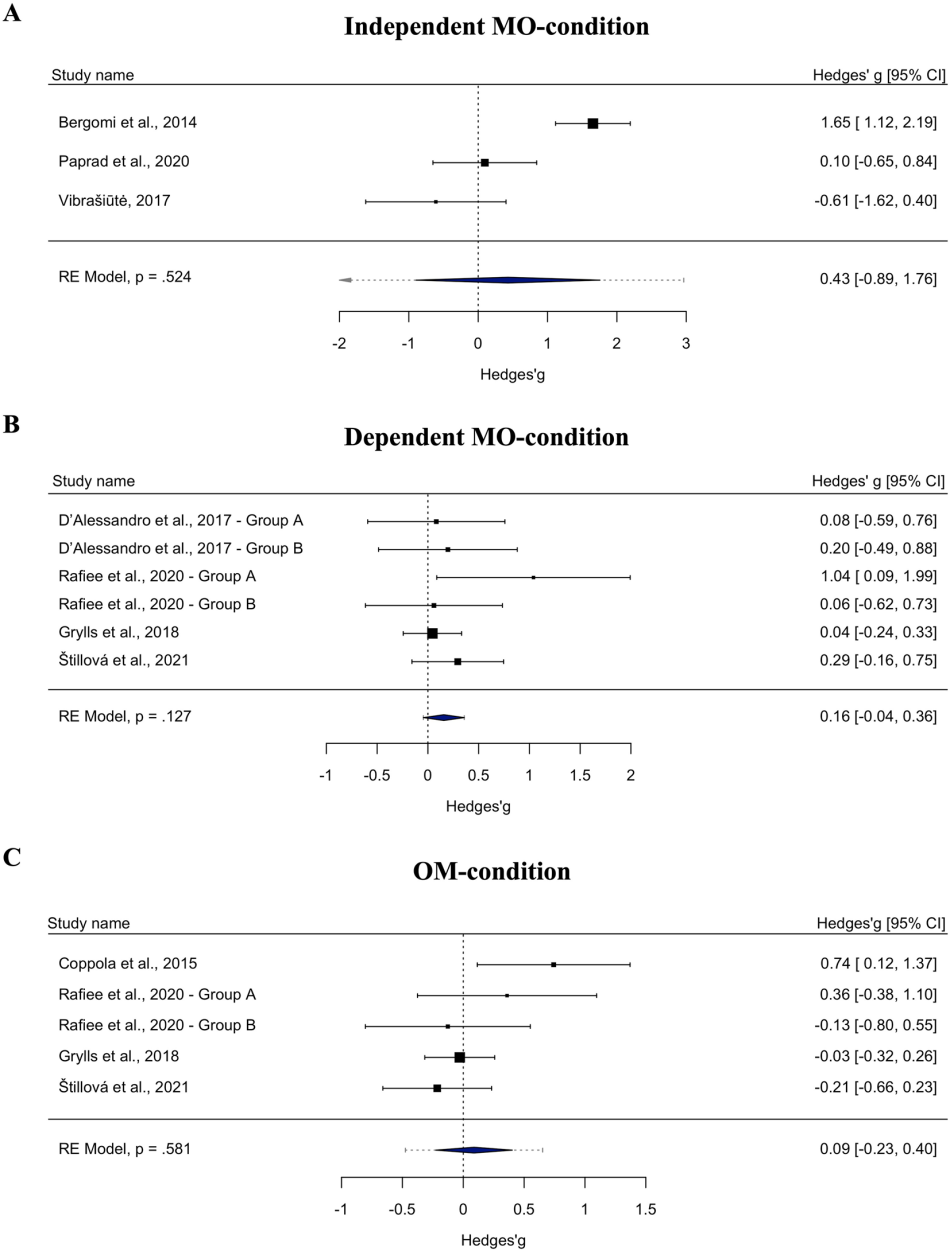
Forest plots for three independent meta-analyses. Effect sizes are provided Hedges’*g* metric with 95% confidence intervals (CI).

We ran three independent random-effects analyses for each condition (see Fig. 2; Table 3). First, the independent MO-condition yielded a non-significant summary effect of *g* = 0.431 (*p* = 0.52, 95% CI [− 0.89, 1.76], *k* = 3), yielding a sign in the expected direction (i.e., favoring listening to Mozart over silence). Although the summary effect was non-trivial in size, the low power did not suggest a meaningful beneficial effect of KV448 on medically relevant outcomes.

**Table 3:** Summary effects for overall and subgroup analyses for three independent meta-analyses.

	Summary effect	*SE*	*p*-value	95% CI	*Q*	τ^2^ (*SE*)	*I*^2^ (%)
Independent MO-condition							
Overall (*k* = 3)	*g* = 0.431	0.676	0.524	[− 0.89, 1.76]	20.485***	1.214 (1.374)	89.44
Dependent MO-condition							
Overall (*k* = 6)	*g* = 0.158	0.103	0.127	[− 0.45, 0.35]	4.384	< 0.001 (0.040)	< 0.001
Measurement method							
Seizure frequency observation (*k* = 4)	*g* = 0.246	0.185	0.182	[− 0.12, 0.60]	3.207	< 0.001 (0.111)	< 0.001
IED (EEG; *k* = 2)	*g* = 0.117	0.124	0.346	[− 0.13, 0.36]	0.836	< 0.001 (0.053)	< 0.001
Funding							
Not reported/no (*k* = 4)	*g* = 0.121	0.110	0.272	[− 0.10, 0.39]	0.897	< 0.001 (0.043)	< 0.001
Yes (*k* = 2)	*g* = 0.490	0.487	0.314	[− 0.46, 1.44]	2.711	0.304 (0.680)	63.12
Sample type							
Adults (*k* = 3)	*g* = 0.332	0.178	0.063	[− 0.02, 0.68]	2.777	< 0.001 (0.107)	< 0.001
Mixed (*k* = 2)	*g* = 0.139	0.245	0.057	[− 0.34, 0.62]	0.054	< 0.001 (0.107)	< 0.001
Seizure type							
Generalized and focal (*k* = 3)	*g* = 0.700	0.126	0.580	[− 0.18, 0.32]	0.165	< 0.001 (0.070)	< 0.001
Not reported/mixed (*k* = 3)	*g* = 0.332	0.178	0.063	[− 0.18, 0.68]	2.777	< 0.001 (0.107)	< 0.001
OM-condition							
Overall (*k* = 5)	*g* = 0.088	0.160	0.581	[− 0.23, 0.40]	7.231	0.057 (0.090)	46.44
Measurement method							
Seizure frequency observation (*k* = 3)	*g* = 0.337	0.262	0.199	[− 0.18, 0.85]	3.406	0.087 (0.207)	41.99
IED-EEG (*k* = 2)	*g* = − 0.083	0.123	0.503	[− 0.32, 0.15]	0.466	< 0.001 (0.052)	< 0.001
Funding							
Not reported/no (*k* = 3)	*g* = 0.115	0.257	0.664	[− 0.39, 0.62]	6.255*	0.145 (0.200)	74.72
Yes (*k* = 2)	*g* = 0.097	0.254	0.704	[− 0.40, 0.59]	0.909	< 0.001 (0.184)	< 0.001
Sample type							
Adults (*k* = 3)	*g* = − 0.075	0.170	0.657	[− 0.40, 0.26]	1.734	< 0.001 (0.094)	95.77
Children (*k* = 2)	*g* = 0.305	0.382	0.425	[− 0.44, 1.05]	4.799*	0.236 (0.421)	79.16
Type of control music							
Other classical (*k* = 2)	*g* = 0.238	0.477	0.618	[− 0.69, 1.17]	5.921**	0.380 (0.647)	83.11
Non-classical/scrambled (*k* = 3)	*g* = 0.003	0.127	0.983	[− 0.24, 0.25]	1.091	< 0.001 (0.074)	< 0.001

Independent MO = independent-groups pretest–posttest designs examining KV448 versus silence^11,27,28^; Dependent MO = one-group pretest–posttest designs examining KV448 versus silence^7,12,25,26^, OM = one-group pretest–posttest designs examining other music versus silence^12,25,26,29^; positive signs indicate a beneficial effect of KV448 (MO) or other music (OM) compared to silence; *k* = number of effect sizes included in the respective condition; IED = interictal epileptic discharges; EEG = electroencephalogram*; SE* = standard error; 95% CI = 95% lower and upper bound of 95% confidence interval; *Q* = Cochran’s *Q* test statistic for heterogeneity; τ^2^ = between-studies variance;* I*^2^ = ratio between true heterogeneity and total observed variation; *p < 0.05; **p < 0.01; ***p < 0.001.

Second, the dependent MO-condition yielded a non-significant trivial summary effect of *g* = 0.158 (*p* = 0.127, 95% CI [− 0.04, 0.36], *k* = 6). This conforms to our above finding of no beneficial influence of KV448 on epilepsy.

Finally, the OM-condition yielded a non-significant trivial summary effect of *g* = 0.088 (*p* = 0.581, 95% CI [− 0.23, 0.40], *k* = 5), showing no benefit of listening to any versus no stimuli on epilepsy.

Sensitivity (i.e., leave-one-out) analyses showed that removing the effect size pertaining to other-reported premature infant pain (Bergomi et al. 2014) from the independent MO-condition leads to a reversal of the summary effect sign, indicating that this effect was solely driven by the inclusion of a single effect. No further summary effect size changes were observed for any other sensitivity analysis results (see Table 4).

**Table 4:** Leave-one-out sensitivity analyses showing summary effects when respective studies were removed from analyses.

Study name	Summary effect	*SE*	*p-*value	95% CI	*Q*	τ^2^	*I*^2^ (%)
Independent MO-condition (*k* = 3)							
Bergomi et al. (2014)	*g* = − 0.171	0.342	0.617	[− 0.84, 0.50]	1.209	0.043	17.30
Paprad et al. (2020)	*g* = 0.565	1.132	0.618	[− 1.65, 2.18]	15.003***	2.393	93.34
Vibrasiute (2017)	*g* = 0.898	0.779	0.249	[− 0.63, 2.43]	10.986**	1.104	90.90
Dependent MO-condition (*k* = 6)							
D’Alessandro et al. (2017)—Group A	*g* = 0.164	0.108	0.128	[− 0.05, 0.38]	4.334	< 0.001	0.01
D’Alessandro et al. (2017)—Group B	*g* = 0.153	0.108	0.155	[− 0.06, 0.36]	4.370	< 0.001	0.01
Rafiee et al. (2020)—Group A	*g* = 0.115	0.105	0.272	[− 0.09, 0.32]	0.926	< 0.001	< 0.001
Rafiee et al. (2020)—Group B	*g* = 0.166	0.108	0.122	[− 0.04, 0.38]	4.297	< 0.001	0.01
Grylls et al. (2018)	*g* = 0.265	0.144	0.066	[− 0.02, 0.55]	3.234	< 0.001	< 0.001
Stillova et al. (2021)	*g* = 0.123	0.115	0.285	[− 0.10, 0.35]	3.940	< 0.001	< 0.001
OM-condition (*k* = 5)							
Coppola et al. (2015)	*g* = − 0.049	0.111	0.662	[− 0.26, 0.17]	1.778	< 0.001	< 0.001
Rafiee et al. (2020)—Group A	*g* = 0.053	0.185	0.773	[− 0.30, 0.42]	6.425	0.074	56.11
Rafiee et al. (2020)—Group B	*g* = 0.146	0.202	0.470	[− 0.25, 0.54]	6.987	0.096	61.65
Grylls et al. (2018)	*g* = 0.161	0.234	0.491	[− 030, 0.62]	6.831	0.120	55.55
Stillova et al. (2021)	*g* = 0.189	0.195	0.332	[− 0.19, 0.57]	5.709	0.072	47.98

*k* = number of effect sizes included in the respective condition; SE = standard error; 95% CI = 95% lower and upper bound of 95% confidence interval; *Q* = Cochran’s *Q* test statistic for heterogeneity; τ^2^ = between-studies variance;* I*^2^ = ratio between true heterogeneity and total observed variation; **p < 0.01; ***p < 0.001.

### Moderator analyses

No nominally statistically significant group differences were identified in any of our analyses, most likely owing to the low power of the available data. Overall and within-subgroup summary effects are provided in Table 3. Continuous moderator effects were examined by means of linear precision-weighted meta-regressions but did not yield any meaningful influences either (see Table 5 for numerical outcomes).

**Table 5:** Numerical outcomes of linear precision-weighted meta-regressions for three independent meta-analyses.

Predictor	*b (SE)*	*R*^2^ (%)	*p-*value	95% CI	*Q*	τ (*SE*)	*I*^2^ (%)
Independent MO-condition (*k* = 3)							
Publication year	− 0.264 (0.276)	< 0.01	0.338	[− 0.80, 0.28]	0.916	1.256 (2.080)	85.42
Age	− 0.023 (0.019)	23.73	0.220	[− 0.06, 0.01]	1.508	0.926 (1.471)	89.07
Percentage of men in samples	0.074 (0.019)	97.08	< 0.001	[0.04, 0.11]	14.867***	0.035 (0.260)	19.27
Duration of exposure	0.002 (0.003)	< 0.01	0.543	[− 0.01, 0.01]	0.371	1.814 (2.849)	90.01
Dependent MO-condition (*k* = 6)							
Publication year	0.072 (0.072)	< 0.01	0.316	[− 0.07, 0.21]	1.006	< 0.001 (0.051)	< 0.001
Age	0.007 (0.007)	< 0.01	0.299	[− 0.01, 0.02]	1.080	< 0.001 (0.064)	< 0.001
Percentage of men in samples	− 0.006 (0.007)	< 0.01	0.411	[− 0.02, 0.01]	0.676	0.003 (0.046)	4.01
Duration of exposure	0.001 (0.001)	< 0.01	0.395	[− 0.01, 0.01]	0.723	< 0.001 (0.073)	< 0.001
OM-condition (*k* = 5)							
Publication year	− 0.123 (0.066)	73.28	0.062	[− 0.25, 0.01]	3.488	0.015 (0.064)	18.65
Age	− 0.007 (0.011)	< 0.01	0.549	[− 0.03, 0.02]	0.360	0.105 (0.152)	57.21
Percentage of men in samples	0.003 (0.015)	< 0.01	0.851	[− 0.03, 0.03]	0.035	0.094 (0.129)	62.38
Duration of exposure	< 0.001 (< 0.001)	100.00	0.023	[< 0.001, < 0.001]	5.188*	< 0.001 (0.045)	< 0.001

Independent MO = independent-groups pretest–posttest designs examining KV448 versus silence^11,27,28^; Dependent MO = one-group pretest–posttest designs examining KV448 versus silence^7,12,25,26^, OM = one-group pretest–posttest designs examining other music versus silence^12,25,26,29^; positive signs indicate a beneficial effect of KV448 (MO) or other music (OM) compared to silence; *k* = number of effect sizes included in the respective condition; *b* = unstandardized regression coefficient; *R*^2^ = proportion of variance in the dependent variable explained by the independent variable; *SE* = standard error; 95% CI = 95% lower and upper bound of 95% confidence interval; *Q* = Cochran’s *Q* test statistic for heterogeneity; τ^2^ = between-studies variance;* I*^2^ = ratio between true heterogeneity and total observed variation; *p < 0.05; ***p < 0.001.

### Publication bias

Publication bias analyses were conducted for published studies only. Sample numbers had to exceed *k* = 2 within analyses for calculating funnel plot-, trim-and-fill, -Egger’s regression-, and PET-PEESE-based methods. Numerical outcomes of all methods applied are provided in Table S3, available at https://osf.io/b8ury.

There was some evidence for publication bias in the independent MO-condition with the selection model approach indicating lacking effect robustness and the excess significance test showing a significantly larger number of published significant effects than would be expectable based on the observed study power. However, *p*-curve-based analysis indicated some evidential value of the data (no funnel plot asymmetry-based methods could be used due to *k* = 2).

**Figure 3:**
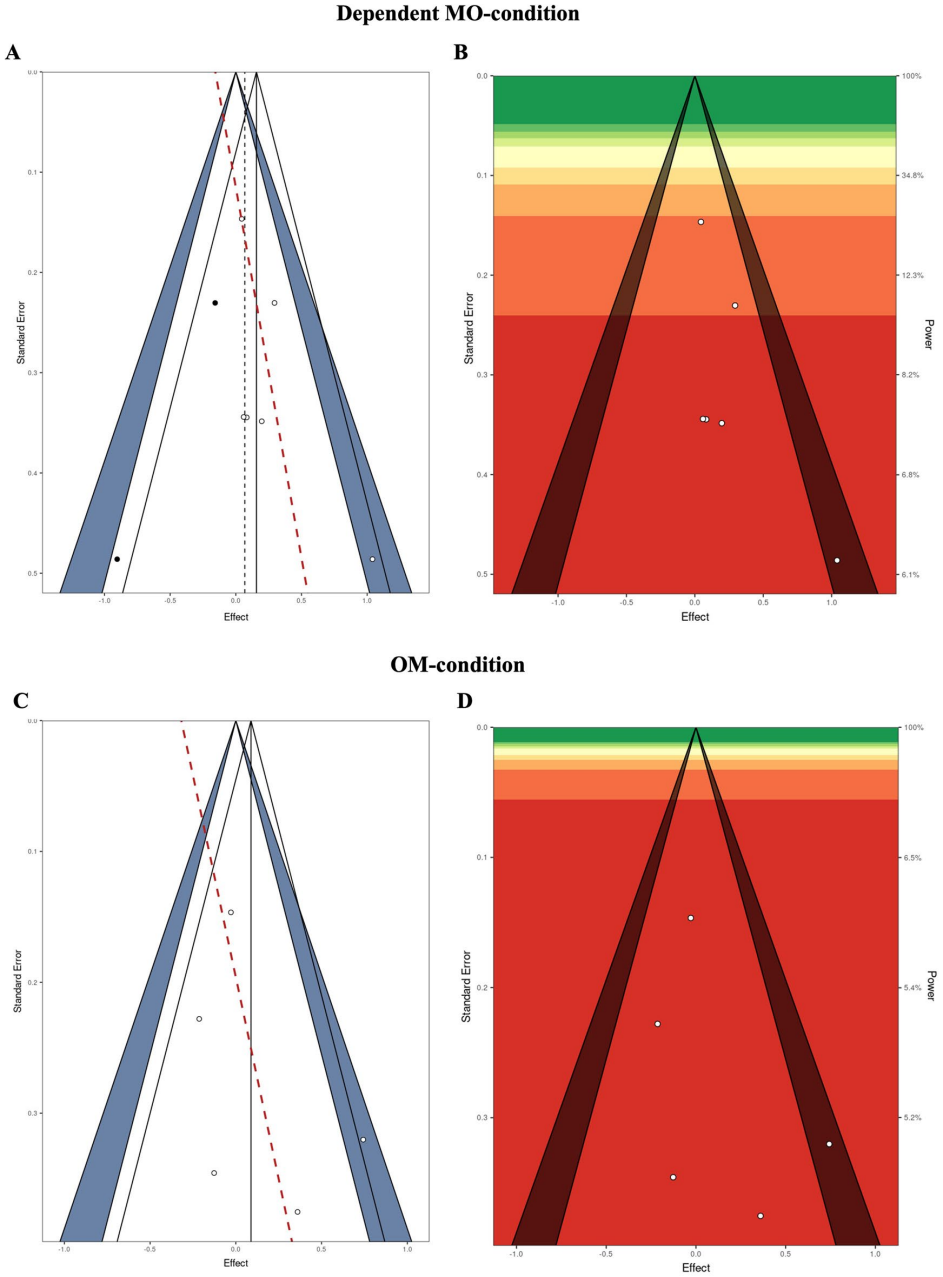
Contour-enhanced funnel plots with imputed trim-and-fill values as well as Egger’s regression line (Panels (**A**) and (**C**) for the dependent MO- and OM conditions) and power-enhanced funnel plots (Panels (**B**) and (**D**) for the dependent MO- and OM conditions) of published sample effect sizes. The dependent MO-condition includes studies with one-group pretest–posttest designs examining exposure to KV448 versus silence^7,12,25,26^. The OM-condition includes studies with one-group pretest–posttest designs examining exposure to other music than KV448 versus silence^12,25,26,29^. Primary study power of effect sizes in segments with cold colors is larger (dark green indicates 90–100 percent power) than those in segments with warmer colors (dark red indicates 0–10 percent power); segments represent 10 percent increments. The highest level of individual study power observed in Panel (**A**) was 15% and 6.5% in Panel (**B**).

Similar results were observed for the dependent MO-condition where both trim-and-fill as well as selection model approaches indicated bias evidence, although in this case *p*-curve-based analyses indicated lacking evidential value of the included data. Visual inspection of contour- and power-enhanced funnel plots indicated funnel plot asymmetry as well as low power of published studies (maximum single study power = 15%; Fig. 3, panels A and B).

Observations for the OM-condition were broadly similar to both other analyses, showing some evidence for publication bias based on selection models and showing considerably underpowered studies in power-enhanced funnel plots (there was no evidence for asymmetry though; maximum single study power = 6.5%; Fig. 3, panels C and D).

It needs to be acknowledged that publication bias detection models typically underperform in presence of small effect numbers^30^. Therefore, it seems surprising that several methods were indicative of bias, although these results need to be taken with a grain of salt. However, the power-related analyses of evidential value in the *p*-curve analyses clearly indicate that the evidential value for any beneficial influence of KV448 in dependent MO-designs is entirely insufficient.

### Specification curve

**Figure 4:**
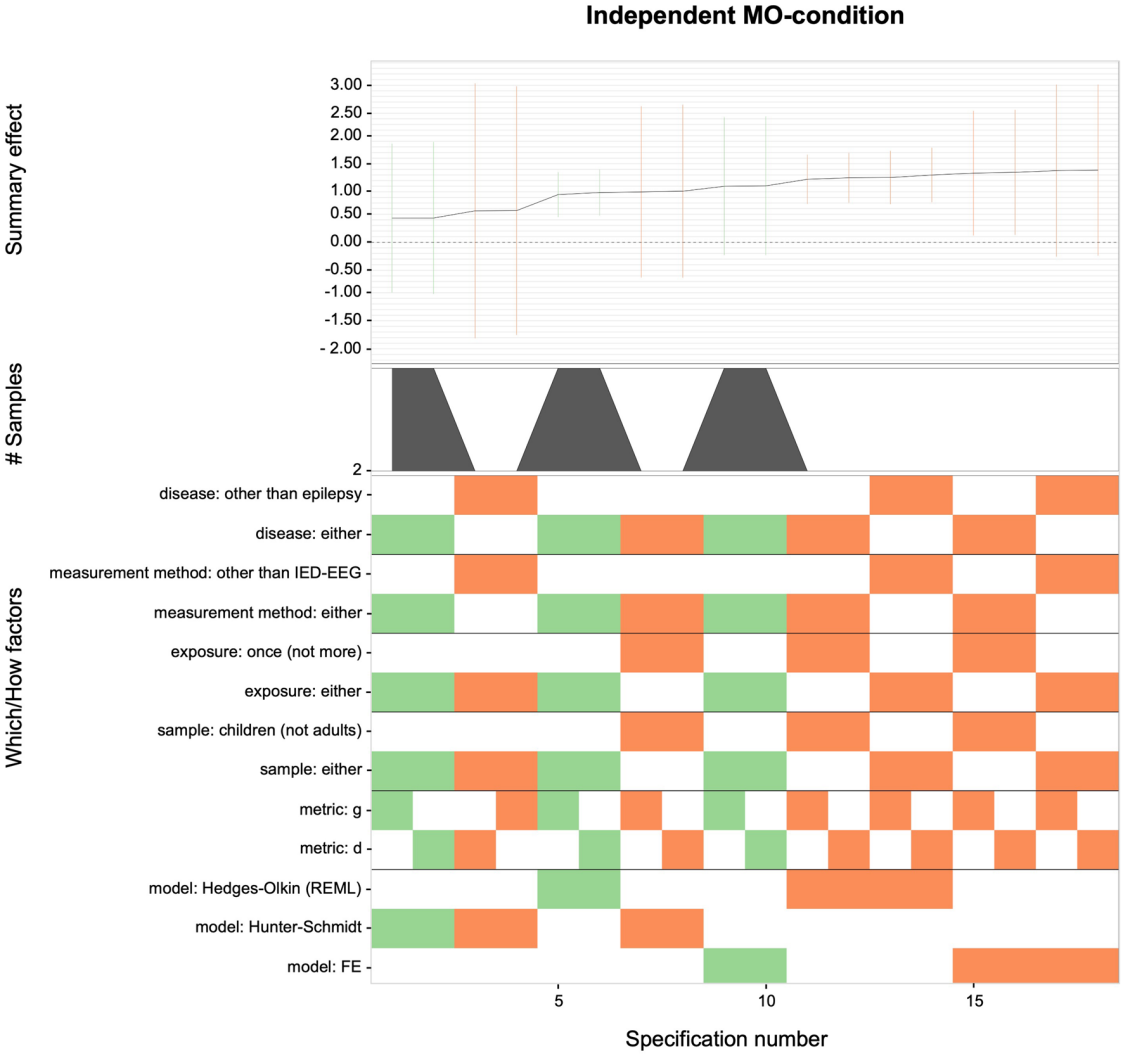
Descriptive meta-analytic specification plot of summary effects from all reasonable specifications for the independent MO-condition. The independent MO-condition includes studies with pretest–posttest designs of independent groups examining exposure to KV448 versus silence^11,27,28^. The bottom panel indicates the “*which*” and “*how*” factors that were included (warmer vs. cooler spectral colors are indicative of lower vs. higher presision of estimates) for the estimated summary effects depicted in the top panel with respective 95% confidence intervals. The center panel indicates the number of samples within the respective subsets.

Figure 4 illustrates that virtually any reasonable specification in the independent MO-condition leads to unprecise estimates (i.e., effects with comparatively wide confidence intervals), which ranged from *g* = 0.40 to *g* = 1.30. The specified summary effects were mostly nominally non-significant (*p* > 0.05), as their 95%-confidence intervals included zero. Here, summary effect estimates were based on a maximum of three studies, whereof only one examined epilepsy. Specifications that included the epilepsy study yielded smaller effect sizes than those that included other medically relevant conditions only.

**Figure 5:**
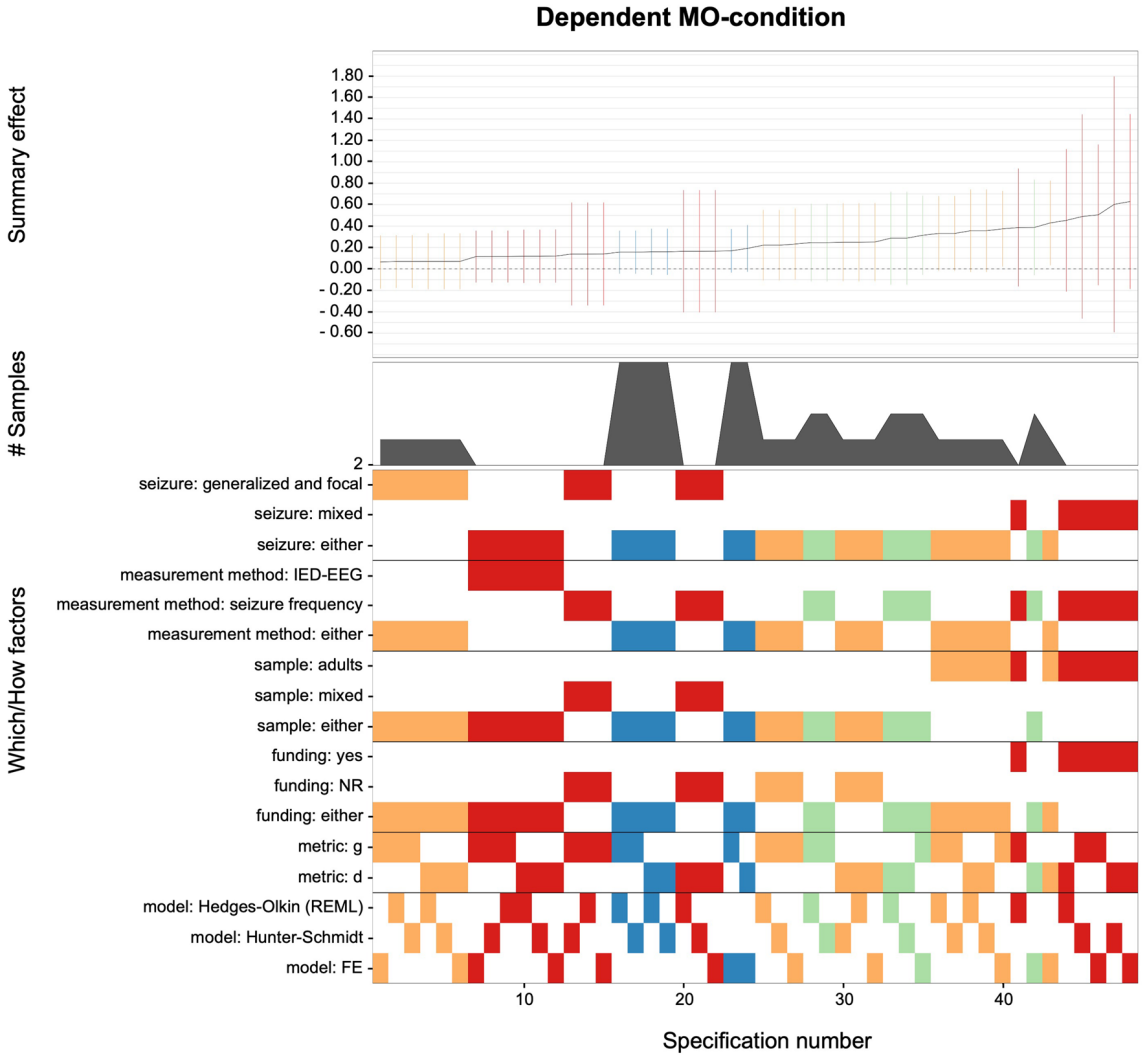
Descriptive meta-analytic specification plot of summary effects from all reasonable specifications for the dependent MO-condition. The dependent MO-condition includes studies with one-group pretest–posttest designs examining exposure to KV448 versus silence^7,12,25,26^. The bottom panel indicates the “*which*” and “*how*” factors that were included (warmer vs. cooler spectral colors are indicative of lower vs. higher presision of estimates) for the estimated summary effects depicted in the top panel with respective 95% confidence intervals. The middle panel indicates the number of samples within the respective subsets.

**Figure 6:**
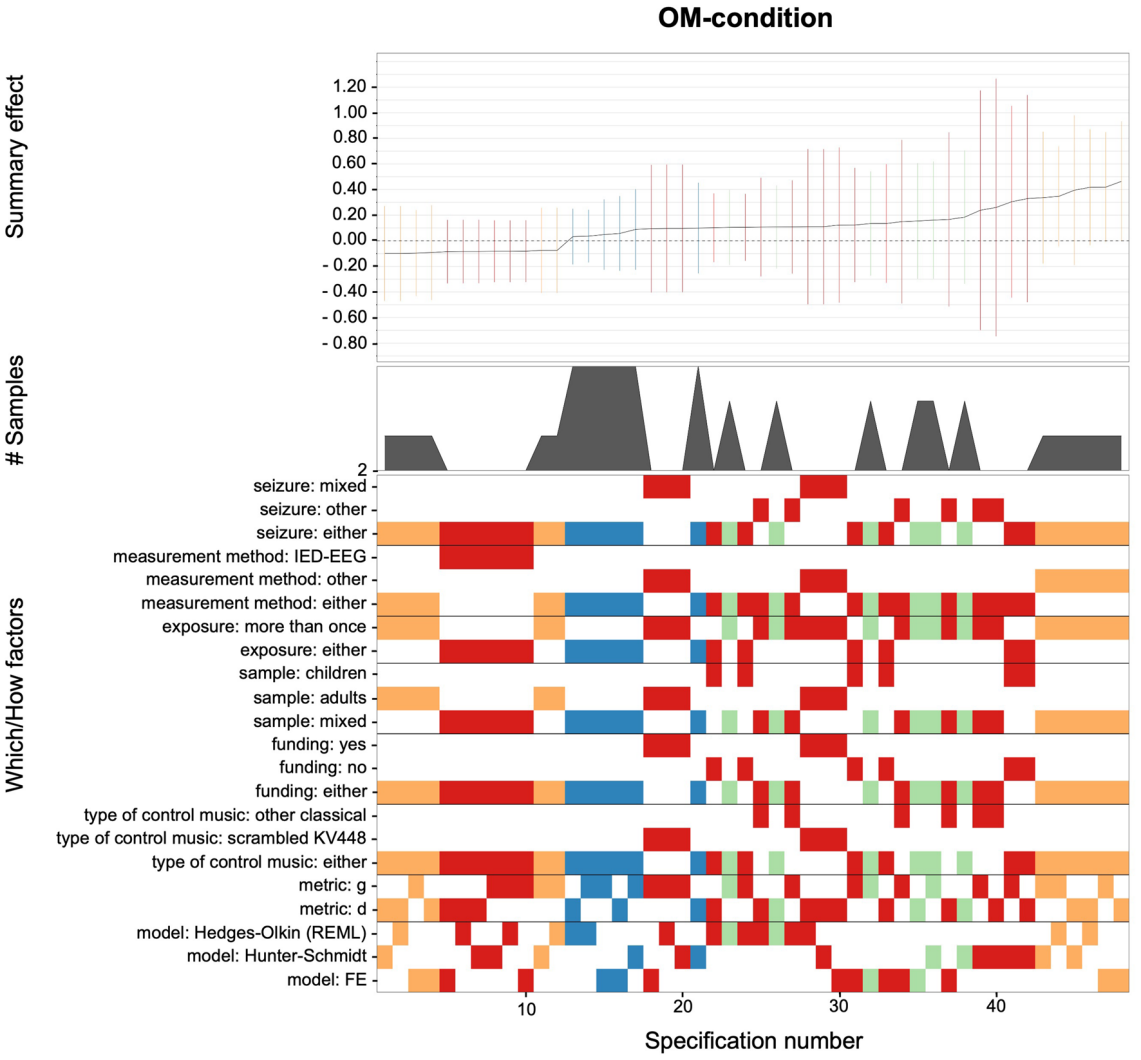
Descriptive meta-analytic specification plot of summary effects from all reasonable specifications for the OM-condition. The OM-condition includes studies with one-group pretest–posttest designs examining exposure to other music than KV448 versus silence^12,25,26,29^. The bottom panel indicates the “*which*” and “*how*” factors that were included (warmer vs. cooler spectral colors are indicative of lower vs. higher presision of estimates) for the estimated summary effects depicted in the top panel with respective 95% confidence intervals. The middle panel indicates the number of samples within the respective subsets.

A similar picture of unprecise effect sizes emerged for all reasonable specifications of the (i) dependent MO-condition (*g* range: 0.08–0.62; see Fig. 5) and (ii) OM-condition (*g* range: − 0.10 to 0.48; see Fig. 6). For both conditions, merely 2 out of 48 specifications yielded nominally significant outcomes, which means that evidence for any beneficial effects of KV448 or other music on epilepsy seems doubtful.

Particularly, the large number of non-significant effects is surprising because, given the inherently large power of meta-analyses, non-significant summary effect sizes are sparse and are typically only observable in absence of any systematic type of intervention.

### Combinatorial meta-analyses

**Figure 7:**
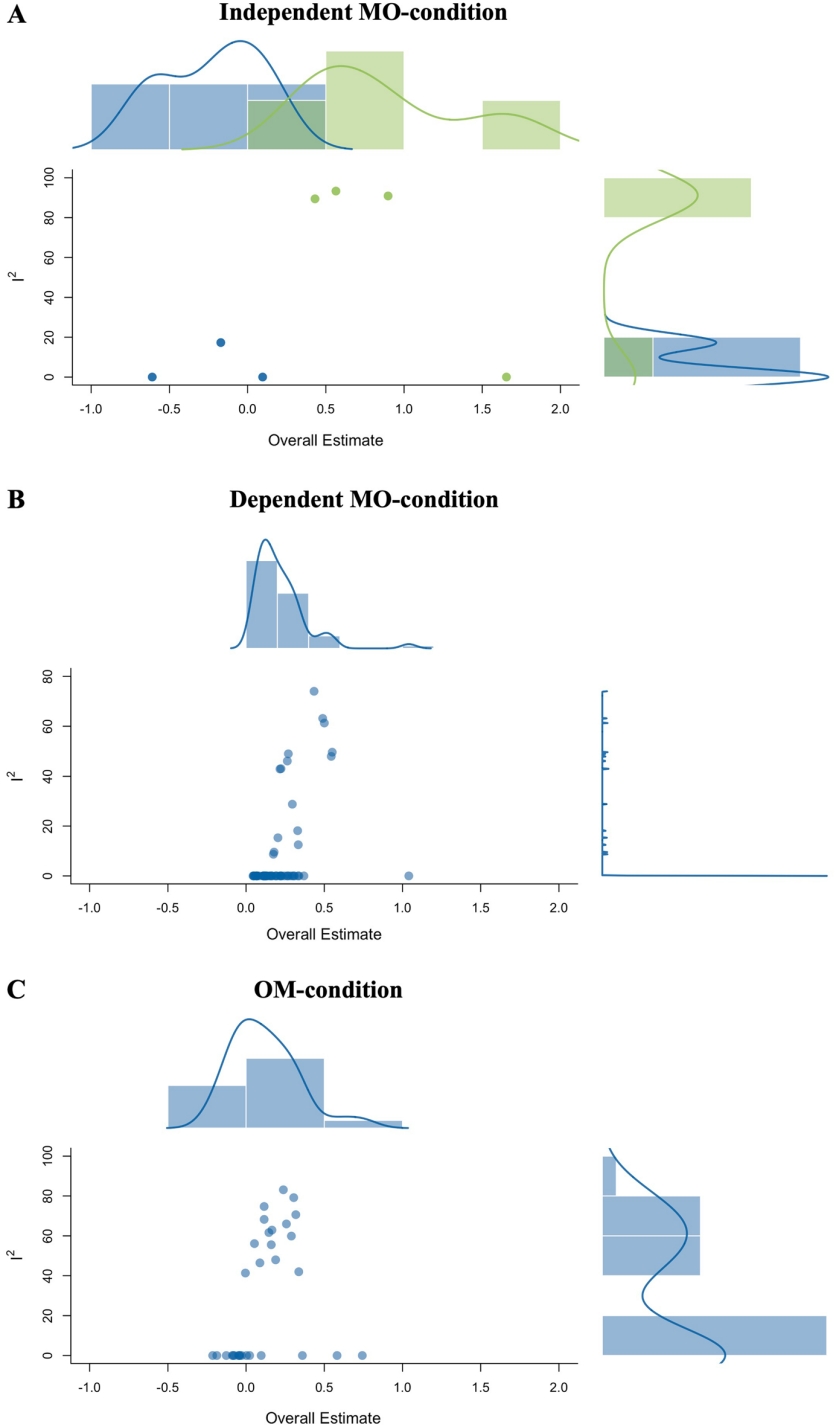
GOSH-plots of all possible combinations for each condition. The independent MO-condition includes studies with pretest–posttest designs of independent groups examining exposure to KV448 versus silence^11,27,28^. The dependent MO-condition includes studies with one-group pretest–posttest designs examining exposure to KV448 versus silence^7,12,25,26^. The OM-condition includes studies with one-group pretest–posttest designs examining exposure to other music than KV448 versus silence^12,25,26,29^. Panel (**A**) shows all 7 possible combinations of *k* = 3 studies included in the independent MO-condition, whereas subset estimations including the study which reported the largest effect size (Bergomi et al. 2014; third-party-reported pain in premature infants) is highlighted in green. Panel (**B**) shows all 63 possible combinations of *k* = 6 studies included in the dependent MO-condition. Panel (**C**) shows all 31 possible combinations of *k* = 5 studies included in the OM-condition. In all conditions, results of combinatorial meta-analyses did not reveal evidence for a salient beneficial effect of either exposure to KV448 or any other kind of music on epilepsy or other medically relevant conditions.

Combinatorial meta-analyses are visualized in Fig. 7 (GOSH plots). In all conditions, results of sampled subsets of all possible combinations did not reveal evidence for any consistent effect of either KV448 or any other kind of music. For the independent MO-condition (Fig. 7, panel A), effect sizes ranged from *g* = − 0.61 to *g* = 1.65. Of note, individual combinations with the study providing the largest effect size^28^ (outcome variable = other-reported pain in premature infants) clearly exerted massive influences on effect strengths as well as heterogeneity. Effect sizes of subsets not including the outlier study ranged from *g* = − 0.60 to *g* = 0.09.

For the dependent MO-condition (Fig. 7, panel B), effect sizes ranged from *g* = 0.04 to *g* = 1.04. For the OM-condition (Fig. 7, panel C), effect sizes ranged from *g* = − 0.21 to *g* = 0.74.

In all conditions, larger effect sizes were associated with higher heterogeneity, indicating that single (uncharacteristic) studies were responsible for (spectacular) hypothesis-conforming effects.

## Discussion

Our present evidence shows that there is only little evidence for any meaningful beneficial effect of listening to Mozart’s sonata KV448 (or any other music) on epilepsy in particular or other medically relevant conditions in general. None of our formal statistical syntheses investigating effects of either Mozart’s sonata KV448 or any other type of music compared to non-musical stimuli yielded any significant summary effects. Although some of the observed effects were non-trivial in terms of strength, examinations of the accumulated study power indicated that the available evidential value was insufficient. These conclusions are rooted in (i) inconsistent and volatile primary study effects, (ii) underpowered primary studies which lead to lacking evidential value of synthesized effects, and (iii) insufficient documentation of the available reports in the published literature which leads to unfounded authority of individual frequently cited studies.

First, there were three RCT-based primary studies (i.e., the gold-standard approach in examining experimental interventions) that investigated influences of KV448 compared to silence on epilepsy or other medically relevant conditions. Although we observed a small-to-moderately-sized summary Mozart effect, our sensitivity analyses and GOSH-plots showed that this effect was driven by a single study that examined influences on third-party-reported pain perceptions of premature infants^28^ which also contributed to substantial increases in between-studies heterogeneity.

Interestingly, both remaining RCTs that reported effects on more objectively operationalizable outcomes showed either evidence for a merely trivial positive effect of KV448 on epilepsy discharges^11^ and even a negative one on blood pressure in stroke patients^27^. This means, that the available RCT-based studies do not support the notion of a specific Mozart effect for epilepsy or other medical conditions.

Analyses of the remaining non-RCT-based studies did not reveal any significant meta-analytical summary effects either. Although some of the observed subgroup-effects were non-trivial in terms of effect strength and conformed to the expected effect direction (i.e., yielding more favorable results for KV448-exposed groups compared to others), non-significance of meta-analytical summary effects indicate substantial power problems of included primary studies. This problem is exacerbated by evidence for some publication bias, which suggests that any observed effects may have been somewhat overestimated within this meta-analytical subset.

The notion of a specific Mozart effect for epilepsy or any other medical conditions seems to be further called into question by our observation of similar largely positive (non-)trivial effects of any other music compared to non-musical stimuli. Again, none of the observed summary effects were significant, thus raising concerns about the power of included primary studies. The bias as well as subset effect-patterns remained essentially the same.

These conclusions are supported by the results of our specification curve and combinatorial analyses which did not indicate meaningful effects of KV448 on medical conditions in general or on epilepsy in particular. Again, the only significant summary effects appeared to be driven by a single non-epilepsy-related study effect^28^ that contributed substantially to the observed between-studies heterogeneity.

Second, sample sizes within included primary studies were small, ranging from *n* = 11–70, which obviously raises concerns about the power to detect any meaningful effects. For instance, a two-group repeated-measures ANOVA design that accounts for possible interactions would require a total sample size of 200 participants to detect a non-trivial effect (i.e., *f* = 0.10 with 80% power and two measurement points at alpha = 0.05). This means that the conceptually most meaningful (i.e., RCT-based) designs in the present analysis were insufficiently powered to detect a non-trivial effect. Even for our largest observed summary effect (i.e., a small-to-moderate *g* = 0.43 in the independent MO-condition) only a single study had sufficient power to detect such an effect^28^. However, this study effect must be considered to represent an outlier, as described above. This interpretation is consistent with our *p*-curve-based analyses which revealed insufficient evidential value of our dependent MO-condition summary effect sizes^24^.

Third, it is concerning that the majority of primary data or even mere summary statistics that document the Mozart effect were unavailable even upon request from the authors. All but one of the studies that had been excluded due to insufficient availability of (summary) data had reported a positive specific Mozart effect in their respective publications. This is particularly concerning because except for one study, all of these studies have been published from 2010 onwards, thus having been published in a time when (i) the awareness of the importance about open science practices should have substantially increased in empirical sciences as evidenced by changes in publication bias detection efforts^19^ and (ii) data sharing practices as well as data availability mandates have been increasingly implemented in Psychology journals in general and those journals that these studies have been published in particular (all journals whose data we could not obtain upon request either mandated or at the very least encouraged authors to share their data).

This is problematic, because despite the uncertain value of non-transparently documented outcomes, the increased attention by both the scientific community and the public on seemingly spectacular outcomes may lead to a perception of (unfounded) authority (i.e., leading readers to assume that a well-established effect exists although there is only little supporting evidence for the finding in question^31^). Novel scientometric measures support this interpretation. For instance, altmetrics for a recently published paper on the Mozart effect on epilepsy indicate considerable readership attention (e.g., within journal performance: percentile 99 of online attention compared to all other articles; among the top 50 of most downloaded papers; editor’s choice collection in chronic diseases), yet no data (or response) were obtainable upon request from the corresponding authors (see, Table S2). We do not mean to suggest, that results from underdocumented findings are necessarily flawed, but their value for answering specific research questions is uncertain. However, we presently observed that (i) most (ostensibly supporting) evidence cannot be evaluated, because numerical results are not reported or unavailable upon request and (ii) the available evidence provides insufficient support for a (specific) Mozart effect for epilepsy.

In all, our meta-analysis shows that there is no meaningful support for a beneficial effect of listening to Mozart’s sonata KV448 on any medically relevant conditions, let alone a specific Mozart effect for epilepsy. Unfounded authority, underpowered studies, and non-transparent reporting appear to be the main drivers of the Mozart effect myth.

## Supplementary Information

Supplementary Information.

## Data Availability

All raw data are provided at https://osf.io/t5wyb and https://osf.io/kjwrn.
