# Supplementary material for: Unfounded authority, underpowered studies, and non-transparent reporting perpetuate the Mozart effect myth: a multiverse meta-analysis
Source: Sci Rep. 2023 Mar 6;13:3175. doi: 10.1038/s41598-023-30206-w (PMC9988829; doi:10.1038/s41598-023-30206-w)
Supplement: Supplementary file 1 — Supplementary Information. [file 41598_2023_30206_MOESM1_ESM.docx]

**Supplementary Materials**

**Table S1.** *Prisma-checklist.*

| **Section and Topic** | **Item #** | **Checklist item** | **Location where item is reported** |
| --- | --- | --- | --- |
| **TITLE** | | |  |
| Title | 1 | Identify the report as a systematic review. | 1 |
| **ABSTRACT** | | |  |
| Abstract | 2 | See the PRISMA 2020 for Abstracts checklist. | 1 |
| **INTRODUCTION** | | |  |
| Rationale | 3 | Describe the rationale for the review in the context of existing knowledge. | 2 |
| Objectives | 4 | Provide an explicit statement of the objective(s) or question(s) the review addresses. | 2 |
| **METHODS** | | |  |
| Eligibility criteria | 5 | Specify the inclusion and exclusion criteria for the review and how studies were grouped for the syntheses. | 3 |
| Information sources | 6 | Specify all databases, registers, websites, organisations, reference lists and other sources searched or consulted to identify studies. Specify the date when each source was last searched or consulted. | 3 |
| Search strategy | 7 | Present the full search strategies for all databases, registers and websites, including any filters and limits used. | 3 |
| Selection process | 8 | Specify the methods used to decide whether a study met the inclusion criteria of the review, including how many reviewers screened each record and each report retrieved, whether they worked independently, and if applicable, details of automation tools used in the process. | 3 |
| Data collection process | 9 | Specify the methods used to collect data from reports, including how many reviewers collected data from each report, whether they worked independently, any processes for obtaining or confirming data from study investigators, and if applicable, details of automation tools used in the process. | 3 |
| Data items | 10a | List and define all outcomes for which data were sought. Specify whether all results that were compatible with each outcome domain in each study were sought (e.g. for all measures, time points, analyses), and if not, the methods used to decide which results to collect. | 3, supp: coding_file |
|  | 10b | List and define all other variables for which data were sought (e.g. participant and intervention characteristics, funding sources). Describe any assumptions made about any missing or unclear information. | 3, supp: coding_file |
| Study risk of bias assessment | 11 | Specify the methods used to assess risk of bias in the included studies, including details of the tool(s) used, how many reviewers assessed each study and whether they worked independently, and if applicable, details of automation tools used in the process. | 3 |
| Effect measures | 12 | Specify for each outcome the effect measure(s) (e.g. risk ratio, mean difference) used in the synthesis or presentation of results. | 4 |
| Synthesis methods | 13a | Describe the processes used to decide which studies were eligible for each synthesis (e.g. tabulating the study intervention characteristics and comparing against the planned groups for each synthesis (item #5)). | 4 |
|  | 13b | Describe any methods required to prepare the data for presentation or synthesis, such as handling of missing summary statistics, or data conversions. | 4 |
|  | 13c | Describe any methods used to tabulate or visually display results of individual studies and syntheses. | 4 |
|  | 13d | Describe any methods used to synthesize results and provide a rationale for the choice(s). If meta-analysis was performed, describe the model(s), method(s) to identify the presence and extent of statistical heterogeneity, and software package(s) used. | 4 |
|  | 13e | Describe any methods used to explore possible causes of heterogeneity among study results (e.g. subgroup analysis, meta-regression). | 4, Table 1 |
|  | 13f | Describe any sensitivity analyses conducted to assess robustness of the synthesized results. | 4 |
| Reporting bias assessment | 14 | Describe any methods used to assess risk of bias due to missing results in a synthesis (arising from reporting biases). | 4, supp: description_publicationbias_  detectiom_methods |
| Certainty assessment | 15 | Describe any methods used to assess certainty (or confidence) in the body of evidence for an outcome. | 4, 5 |
| **RESULTS** | | |  |
| Study selection | 16a | Describe the results of the search and selection process, from the number of records identified in the search to the number of studies included in the review, ideally using a flow diagram. | 7 |
|  | 16b | Cite studies that might appear to meet the inclusion criteria, but which were excluded, and explain why they were excluded. | Table S2 |
| Study characteristics | 17 | Cite each included study and present its characteristics. | Table 2 |
| Risk of bias in studies | 18 | Present assessments of risk of bias for each included study. | 7 |
| Results of individual studies | 19 | For all outcomes, present, for each study: (a) summary statistics for each group (where appropriate) and (b) an effect estimate and its precision (e.g. confidence/credible interval), ideally using structured tables or plots. | 7, Figure 2 |
| Results of syntheses | 20a | For each synthesis, briefly summarise the characteristics and risk of bias among contributing studies. | 7 |
|  | 20b | Present results of all statistical syntheses conducted. If meta-analysis was done, present for each the summary estimate and its precision (e.g. confidence/credible interval) and measures of statistical heterogeneity. If comparing groups, describe the direction of the effect. | 7, Figure 2, Table 3 |
|  | 20c | Present results of all investigations of possible causes of heterogeneity among study results. | 7, Table 3 |
|  | 20d | Present results of all sensitivity analyses conducted to assess the robustness of the synthesized results. | 7, Table 3 - 5 |
| Reporting biases | 21 | Present assessments of risk of bias due to missing results (arising from reporting biases) for each synthesis assessed. | 8, Figure 3, supp. Table S3 |
| Certainty of evidence | 22 | Present assessments of certainty (or confidence) in the body of evidence for each outcome assessed. | 9, Figure 4 - 7 |
| **DISCUSSION** | | |  |
| Discussion | 23a | Provide a general interpretation of the results in the context of other evidence. | 14-15 |
|  | 23b | Discuss any limitations of the evidence included in the review. | 14-15 |
|  | 23c | Discuss any limitations of the review processes used. | 14-15 |
|  | 23d | Discuss implications of the results for practice, policy, and future research. | 14-15 |
| **OTHER INFORMATION** | | |  |
| Registration and protocol | 24a | Provide registration information for the review, including register name and registration number, or state that the review was not registered. | 3 |
|  | 24b | Indicate where the review protocol can be accessed, or state that a protocol was not prepared. | 3 |
|  | 24c | Describe and explain any amendments to information provided at registration or in the protocol. | 3 |
| Support | 25 | Describe sources of financial or non-financial support for the review, and the role of the funders or sponsors in the review. | - |
| Competing interests | 26 | Declare any competing interests of review authors. | 16 |
| Availability of data, code and other materials | 27 | Report which of the following are publicly available and where they can be found: template data collection forms; data extracted from included studies; data used for all analyses; analytic code; any other materials used in the review. | 3 |

*From:*  Page MJ, McKenzie JE, Bossuyt PM, Boutron I, Hoffmann TC, Mulrow CD, et al. The PRISMA 2020 statement: an updated guideline for reporting systematic reviews. BMJ 2021;372:n71. doi: 10.1136/bmj.n71

**Table S2.** Excluded studies’ characteristics.

| Reference | Sample size | Sample type | Disease | Conclusion | Journal | Exclusion reason | Data availability |
| --- | --- | --- | --- | --- | --- | --- | --- |
| Attanasio et al. (2012) | 62 | Adults | Tinnitus | Significant positive effect on the sound intensity of tinnitus after a single exposure to the KV448. | Acta Oto-Laryngologica | - Statistical information to calculate effect sizes was unavailable - One-group pre-post design counterbalanced, no wash-out period | Not reported and no author response. |
| Bedetti et al. (2019) | 1 | Adult | Epilepsy | Significant reduction of epileptic discharges and the frequency of seizures. | Psychiatria Danubina | - Case study - Patient was affected by profound intellectual disability, autism spectrum disorder, intermittent explosive disorder, drug-resistant epilepsy, microcephaly, facial and limbs dysmorphisms | - |
| Bodner et al. (2012) | *n* KV448 = 25  *n* control = 11 | Mixed | Epilepsy | Significant reduction of seizures in subjects with a range of epilepsy and seizure types, in some cases complete cessation of seizures after exposure to KV448. The study was designed as an RCT. | PLOS one | - Statistical information to calculate effect sizes was unavailable | Not reported and no author response. |
| Coppola et al. (2017) | *n* KV448 = 9  *n* control = 10 | Children | Epilepsy | Music therapy seems to be an effective treatment for patients with refractory epileptic seizures in childhood, even though the KV448 was not identified as more effective than other Mozart (i.e., other classical) music. The study was designed as an RCT. | Epilepsy & Behavior | - Only identified study with KV448 vs. other music condition, i.e., was not usable in the present meta-analysis | Summary data provided upon request. |
| Hughes et al. (1998) | 29 | Mixed | Epilepsy | Significant reduction of epileptiform activity in 23 of 29 patients, even if they were in coma, with status epilepticus, or with periodic lateralized epileptiform discharges. | Clinical EEG and Neuroscience | - Statistical information to calculate effect sizes was unavailable - One-group design | No contact information available. |
| Hughes et al. (1999) | 1 | Children | Epilepsy | Significant reduction of seizure frequency, generalized bilateral spike and wave complex over a 24-hour period while exposed to KV448 for ten minutes every hour during wakefulness. | Clinical Electroencephalography | - Case study - Patient suffered from Lennox-Gastaut Syndrome | - |
| Kuester et al. (2010) | 1 | Adults | Epilepsy | Reduction of epileptic discharges, recovering from coma after five days of exposure to KV448. | Epilepsy & Behavior | - Case study - Patient had severe brain trauma | - |
| Lahiri & Ducan (2007) | 1 | Adults | Epilepsy | Complete cessation of secondarily generalized tonic-colonic seizures after three months of listening to Mozart’s music for 45 minutes a day. | Epilepsy & Behavior | - Case study - Self-report of the patient | - |
| Lin et al. (2010) | t0_:_ 58  t1_:_ 46 | Children | Epilepsy | Reduction of epileptiform discharges in children with different seizure types after listening to KV448 once. No decrease in epileptiform discharge was observed when a computerized string  version of the KV448 was used. | Epilepsy Research | - Statistical information to calculate effect sizes was unavailable - One-group design | Data requested, data unavailable. |
| Lin et al. (2011a) | 11 | Children | Epilepsy | After listening to KV448 once a day for six month, two patients were seizure free, six had a significant reduction in seizure frequency and in three patients a minimal or no effect was observed. | Epilepsy & Behavior | - Statistical information to calculate effect sizes was unavailable - One-group design | Data requested, data unavailable. |
| Lin et al. (2011b) | 18 | Children | Epilepsy | Long-term listening to KV448 decreases epileptiform discharge frequency. Seizure frequency was not measured, since all patients were seizure free for six months prior the study. | Epilepsy & Behavior | - Statistical information to calculate effect sizes was unavailable - One-group design | Data requested, data unavailable. |
| Lin et al. (2012) | *N* t0 = 39  *N* KV545 t1= 34  *N* KV448 t1 = 33 | Children |  | Significant decrease in epileptiform discharges in children after listening to KV448 and KV545. | Evidence-Based Complementary and Alternative Medicine | - Statistical information to calculate effect sizes was unavailable - Inaccurate reporting with regard to dropouts and group sizes - One-group design counterbalanced, wash-out period one week only | Data requested, data unavailable. |
| Lin et al. (2013) | *n* KV448 t0 = 41  *n* KV448 t1 = 34  *n* KV545 t0 = 23  *n* KV545 t1 = 19 | Children | Epilepsy | Significant decrease of epileptiform discharges in children with epilepsy. During music (KV448 / KV545), an increased parasympathetic tone was observed. | Clinical Neurophysiology | - Statistical information to calculate effect sizes was unavailable - Inaccurate reporting with regard to dropouts and group sizes | Data requested, data unavailable. |
| Lin et al. (2014) | *n* KV448 t0= 22  *n* KV448 t1 = 11  *n* KV448 t2 = 10  *n* KV448 t3 = 8  *n* control t0= 24  *n* control t1= 10 | Children | Epilepsy | Significant reduction of seizure recurrence rate and epileptiform discharged in children with first unprovoked seizures. | BMC  Complementary Medicine and Therapies | - Statistical information to calculate effect sizes was unavailable - Inaccurate reporting with regard to dropouts and group sizes - Patients had experienced only a single seizure (unclear, if any of them experienced seizures ever again afterwards) | Data requested, data unavailable. |
| Ren et al. (2019) | *n* KV448 = 11  *n* control = 5 | Children | Epilepsy | Significant reduction of seizure recurrence rate and epileptiform discharged in children with first unprovoked seizures. | BMC –  Complementary Medicine and Therapies | - Statistical information to calculate effect sizes was unavailable | Data requested, data unavailable. |
| Quon et al. (2021) | *n*1 = 8  *n*2 = 8 | Adults | Epilepsy | Reduced interictal epileptiform discharges during the original version of KV448 after at least 30 seconds of exposure. | Scientific Reports | - Statistical information to calculate effect sizes was unavailable | Not reported and no author response. |
| Turner et al. (2004) | t0_:_ = 4  t1_:_ = 2 / 1 | Children | Epilepsy | Significant decreases in interictal epileptiform discharges after exposure to KV448. No Effect for Beethoven’s *Für Elise*. | Epilepsy & Behavior | - Statistical information to calculate effect sizes was unavailable - Results from only one participants were reported | - |
| Ziv et al. (2022) | *n* KV448 = 23  *n* control group 1 = 22  *n* control group 2 = 25 | Children | Bronchiolitis | No significant difference in the severity score of children hospitalized with  bronchiolitis after listening to the KV448. | Acta Pediatrica | - A patient could participate in the study more than once, i.e., the study design is unsuitable | Not reported and no author response. |

*Note.* Upper case *N* means that one sample underwent different conditions; lower case *n* means that different samples underwent different conditions; t: time of measurement.

**References**

Attanasio, G., Cartocci, G., Covelli, E., Ambrosetti, E., Martinelli, V., Zaccone, M., ... & Cacciafesta, M. (2012). The Mozart effect in patients suffering from tinnitus. *Acta oto-laryngologica*, *132*(11), 1172-1177. <https://doi.org/10.3109/00016489.2012.684398>

Bedetti, Principi, M., Di Renzo, A., Muti, M., Frondizi, D., Piccirilli, M., D'Alessandro, P., Marchiafava, M., Baglioni, A., Menna, M., Gubbiotti, M., & Elisei, S. (2019). The Effect of Mozart's music in severe epilepsy: Functional and morphological features. *Psychiatria Danubina*, *31*(3), 467–474.

Bodner, M., Turner, R. P., Schwacke, J., Bowers, C., & Norment, C. (2012). Reduction of seizure occurrence from exposure to auditory stimulation in individuals with neurological handicaps: A randomized controlled trial. PLOS ONE, 7(10), e45303. <https://doi.org/10.1371/journal.pone.0045303>

Coppola, G., Operto, F. F., Caprio, F., Ferraioli, G., Pisano, S., Viggiano, A., & Verrotti, A. (2018). Mozart's music in children with drug-refractory epileptic encephalopathies: comparison of two protocols. *Epilepsy & Behavior*, *78*, 100-103. <https://doi.org/10.1016/j.yebeh.2017.09.028>

Hughes, J. R., Daaboul, Y., Fino, J. J., & Shaw, G. L. (1998). The “Mozart effect” on epileptiform activity. *Clinical Electroencephalography*, *29*(3), 109-119. <https://doi.org/10.1177/155005949802900301>

Hughes, J. R., Fino, J. J., & Melyn, M. A. (1999). Is there a chronic change of the “Mozart effect” on epileptiform activity? A case study. *Clin Electroencephalogr*, *30*(2), 44-45.
<https://doi.org./10.1177/155005949903000204>

Kuester, G., Rios, L., Ortiz, A., & Miranda, M. (2010). Effect of music on the recovery of a patient with refractory nonconvulsive status epilepticus. *Epilepsy & Behavior*, *18*(4), 491-493. <https://doi.org/10.1016/j.yebeh.2010.06.001>

Lahiri, N., & Duncan, J. S. (2007). The Mozart effect: encore. *Epilepsy & Behavior*, *11*(1), 152-153. <https://doi.org/10.1016/j.yebeh.2007.04.017>

Lin, L. C., Lee, W. T., Wu, H. C., Tsai, C. L., Wei, R. C., Jong, Y. J., & Yang, R. C. (2010). Mozart K. 448 and epileptiform discharges: effect of ratio of lower to higher harmonics. *Epilepsy research*, *89*(2-3), 238-245.
<https://doi.org/10.1016/j.eplepsyres.2010.01.007>

Lin, L. C., Lee, W. T., Wang, C. H., Chen, H. L., Wu, H. C., Tsai, C. L., ... & Yang, R. C. (2011a). Mozart K. 448 acts as a potential add-on therapy in children with refractory epilepsy. *Epilepsy & Behavior*, *20*(3), 490-493. <https://doi.org/10.1016/j.yebeh.2010.12.044>

Lin, L. C., Lee, W. T., Wu, H. C., Tsai, C. L., Wei, R. C., Mok, H. K., ... & Yang, R. C. (2011b). The long-term effect of listening to Mozart K. 448 decreases epileptiform discharges in children with epilepsy. *Epilepsy & Behavior*, *21*(4), 420-424.
<https://doi.org/10.1016/j.yebeh.2011.05.015>

Lin, L. C., Lee, M. W., Wei, R. C., Mok, H. K., Wu, H. C., Tsai, C. L., & Yang, R. C. (2012). Mozart k. 545 mimics mozart k. 448 in reducing epileptiform discharges in epileptic children. *Evidence-Based Complementary and Alternative Medicine*, *2012*.
<https://doi.org/10.1155/2012/607517>

Lin, L. C., Chiang, C. T., Lee, M. W., Mok, H. K., Yang, Y. H., Wu, H. C., ... & Yang, R. C. (2013). Parasympathetic activation is involved in reducing epileptiform discharges when listening to Mozart music. *Clinical Neurophysiology*, *124*(8), 1528-1535.
<https://doi.org/10.1016/j.clinph.2013.02.021>

Lin, L. C., Lee, M. W., Wei, R. C., Mok, H. K., & Yang, R. C. (2014a). Mozart K. 448 listening decreased seizure recurrence and epileptiform discharges in children with first unprovoked seizures: a randomized controlled study. *BMC complementary and alternative medicine*, *14*(1), 1-8. <https://doi.org/10.1186/1472-6882-14-17>

Quon, R. J., Casey, M. A., Camp, E. J., Meisenhelter, S., Steimel, S. A., Song, Y., ... & Jobst, B. C. (2021). Musical components important for the Mozart K448 effect in epilepsy. *Scientific reports*, *11*(1), 1-13. <https://doi.org/10.1038/s41598-021-95922-7>

Ren, H., Jiang, X., Xu, K., Zou, L., Wang, L., Lu, C., ... & Chen, W. (2019, October). Evaluation of the effects of mozart music on cerebral hemodynamics in preterm infants. In *2019 IEEE Biomedical Circuits and Systems Conference (BioCAS)* (pp. 1-4). IEEE.
<https://doi.org/10.1109/BIOCAS.2019.8919100>

Turner, R. P. (2004). The acute effect of music on interictal epileptiform discharges. *Epilepsy & Behavior*, *5*(5), 662-668. <https://doi.org/10.1016/j.yebeh.2004.07.003>

Ziv, N., Zagon‐Rogel, M., Waisman, Y., Rom, E., Attias, J., & Krause, I. (2022). The effect of environmental Noise Isolation on Bronchiolitis Severity in Hospitalized Children. *Acta Paediatrica*. <https://doi.org/10.1111/apa.16437>

**S3. Description of publication bias detection methods**

***Funnel plot (Light & Pillemer, 1984)***

Funnel plots were visually inspected to examine the relationship between study precision and effect strength (Borenstein, 2021). It is expected, that less precise studies scatter considerably at the bottom of the plot, while more precise studies scatter less at the top due to smaller error variances of larger studies (Harrer et al., 2021). In absence of bias, distributions are symmetrical and shaped like an inverted funnel that is centered around the summary effect size. In the presence of bias, these distributions are non-symmetrical because unprecise estimates (i.e., those with large sampling errors) remain unpublished (Borenstein et al., 2021). In power-enhanced funnel plots (Kossmeier et al., 2020), the study-level power is visualized by different colors (i.e., warmer colors indicating lower and colder colors indicating larger study power).

***Trim-and-fill (Duval & Tweedie, 2000)***

In the trim-and-fill method (Duval & Tweedie, 2000), the number of study effects on the left and right of the summary effect size estimate are ranked according to their strength and subsequently compared by means of a Wilcoxon test (i.e., akin to the idea of assessing funnel plot asymmetry). In cases of asymmetry, the smallest studies of the funnel plot get removed in an iterative procedure, in which each removal is followed by a summary effect reestimation and asymmetry reassessment (Borenstein et al., 2021). Once no more asymmetry is detected, all trimmed study effects are reentered and the ostensibly missing effects are added around the reestimated study effect (Duval & Tweedie, 2000). We assumed bias if the difference between the meta-analytic summary effects and the adjusted estimate exceeds 20% of the summary effect in either direction following current recommendations (Siegel et al., 2022). Because a minimum of *k* = 3 studies is required for its application, presently the trim-and-fill method was conducted only for the dependent MO-condition as well as the OM-condition (*p* < .10 is assumed to be indicative of bias).

***Sterne and Egger’s regression*** ***(Egger et al., 1997; Sterne & Egger, 2005)***

Sterne and Egger’s regression corresponds to a linear regression of the effect size estimate on its standard error (i.e., the precision), weighted by the inverse variance of the effect estimate.

If the intercept differs significantly from zero, Sterne and Egger’s tests indicate funnel plot asymmetry, which may be indicative of dissemination bias or small-study effects (*p* < .10 is assumed to be indicative of bias; Harrer et al., 2021).

***Rank correlation method (Begg & Mazumdar, 1994)***

The rank correlation approach is based on a non-parametric correlation of the standardized effect size and the variance of the study-specific effect estimate (by the means of Kendall’s τ^2^; Schwarzer & Rücker, 2010). In absence of bias, no systematic association should be observable between study precision and effect strength (i.e., *p* < .10 is assumed to be indicative of bias; Siegel et al., 2022).

***PET-PEESE (Stanley & Doucouliagos, 2014)***

PET-PEESE can be viewed as an extension of Sterne and Egger’s test and comprises two regressions (Cooper et al., 2019). In the first one, a weighted regression of the effect size on the standard error with an intercept indicating the true effect if the standard error equals to zero represents the precision-effect test (PET; Stanley & Doucouliagos, 2014). If the intercept is significantly different from zero in PET, PEESE (precision-effect-estimate with standard error) is conducted (i.e., a weighted regression of the effect size on the squared standard error and its intercept is interpreted as an effect size estimate; Cooper et al., 2019). Evidence for bias is typically assumed if the difference between the meta-analytic summary effects and the adjusted estimate exceeds 20% of the summary effect in either direction (Siegel et al., 2022). Simulation studies indicate possible limitations of PET-PEESE in presence of small study numbers, small within-study sample sizes, and large heterogeneities (Carter et al., 2019).

***Selection model approach (Vevea & Woods, 2005)***

We used the selection model approach by Vevea & Woods (2005), which is based on four different study weight functions for *p*-values. By the means of this approach we assumed that effect sizes were selected either according to moderate vs. severe and one- vs. two tailed criteria (Pietschnig et al., 2015). In this vein, results would be indicative of publication bias if resulting summary effect estimates differed substantially (i.e., exceeding 20%) between the corrected and the uncorrected estimates (Siegel et al., 2022).

***Test of Excess of Significance (Ioannidis & Trikalinos, 2007)***

Based on the Test of Excess Significance we evaluate whether there is an excess of studies with statistically significant results, by comparing the expected number of significant studies (based on the assumption that the estimated summary effect corresponds to the true effect and can consequently be used to calculate primary study power) and the observed number of included significant studies (Ioannidis & Trikalinos, 2007).

***p-curve (Simonsohn et al., 2014), p-uniform (van Assen et al., 2015), and p-uniform* (van Aert & van Assen, in press)***

*p*-curve is a diagnostic tool that assesses evidence for the presence of both publication bias as well as *p*-hacking and illustrates the distribution of published statistically significant *p* values (i.e., *p* < .05) within a given study set (Harris et al., 2021). The shape of the *p*-curve depends on both the evidential value and the sample size of the included studies. Assuming the null hypothesis that small and large *p*-values are equally likely, a uniformly distributed *p*-curve is expected, indicating no evidential value. Under the alternative hypothesis, however, smaller *p*-values are more likely to occur than larger ones, thus leading to a right-skewed distribution of (significant) *p*-values (i.e., this indicates the existence of a true effect; Cooper et al., 2021). Right-skewness is tested by means of binomial tests, which compares the distribution of *p*-values > .025 with the distribution of *p*-values < .025. In order to avoid a loss of information, since binomial test require a dichotomization of the in fact continuous *p*-values, additional continuous test (*Stouffer method*) are performed (Harris et al., 2021). Moreover, by means of *p*-curve, it is possible to assess the evidential value of the available studies by comparing the observed distribution with a conditional *p*-value distribution at 33% power. In cases where both empirical and theoretical conditional distributions do not differ significantly, it must be assumed that the available evidential value is insufficient to assume a valid true effect.. In presence of a left-skewed curve, it must be assumed that *p*-hacked results confound the published evidence (Cooper et al., 2021).

Both *p*-uniform and *p*-uniform* are based on similar ideas as the *p*-curve method, but the former methods use different approaches to define the fit to the uniform distribution (Cooper et al., 2021). The effect estimation in *p*-uniform* is based on *p*-values of both significant and non-significant studies (Harris et al., 2021).

By means of all three methods, summary effect sizes can be estimated with published significant (*p*-curve and *p*-uniform) or all *p*-values (*p*-uniform*). Furthermore, *p*-uniform and *p*-uniform* provide a means to calculate confidence intervals. Of note, it should be considered that *p*-value based methods for detecting publication bias tend to overestimate the effect size of the evidential value (Cooper et al., 2021).

**Table S3.** *Numeric outcomes of publication bias detection methods.*

| Method | Independent MO-condition  (*k* = 2) | Dependent MO-condition  (*k* = 6) | OM condition  (*k* = 5) |
| --- | --- | --- | --- |
| Trim-And-Fill | - | adjusted estimate = 0.068  unadjusted estimate = 0.157  56% change | adjusted estimate = 0.088  unadjusted estimate = 0.088  0% change |
| Rank correlation | - | τ = 0.60 *p* = .136 | τ = 0.40 *p* = .483 |
| Egger’s regression | - | *Z* = 1.31  *p* = .190 | *Z* = 0.982  *p* = .326 |
| PET-PEESE | - | PET  intercept = -0.156  *p* = .503  PEESE  intercept = -0.014  *p* = .914 | PET  intercept = -0.317  *p* = .477  PEESE  intercept = -0.151  *p* = .556 |
| Selection Model | moderate one tailed: 51%  severe one tailed: 183%  moderate two tailed: 7%  severe two tailed: 15% | moderate one tailed: 29%  severe one tailed: 66%  moderate two tailed: 13%  severe two tailed: 28% | moderate one tailed: 129%  severe one tailed: 302%  moderate two tailed: 15%  severe two tailed: 32% |
| Test of Excess Significance | *p* = .035 | *p* = .458 | *p* = .257 |
| *p*-curve | continuous tests yielded *p*’s < .001 for evidential value indication and *p*’s >.999 for p-hacking indication;  estimated statistical power: 99% (*k* = 1) | continuous tests yielded *p =* .449 and *p =* .699 for evidential value indication and *p* = .429 and *p =* .692 for p-hacking indication;  estimated statistical power: 13% (*k* = 1) | - |
| *p*-uniform | *Z* = > 0.001  *p* = .5 | - | - |
| *p*-uniform* | Z = 0.323  *p* = .851 | *Z* = 0.067  *p* = .410 | *Z* = 0.021  *p* = .989 |

*Note.* NA = method was not feasible.

**References**

Begg, C. B., & Mazumdar, M. (1994). Operating characteristics of a rank correlation test for publication bias. *Biometrics*, 1088-1101. <https://doi.org/10.2307/2533446>

Borenstein, M., Hedges, L. V., Higgins, J. P., & Rothstein, H. R. (2021). Introduction to meta-analysis. *John Wiley & Sons*.

Carter, E. C., Schönbrodt, F. D., Gervais, W. M., & Hilgard, J. (2019). Correcting for Bias in Psychology: A Comparison of Meta-Analytic Methods. *Advances in Methods and Practices in Psychological Science*, *2*(2), 115-144. <https://doi.org/10.1177/2515245919847196>

Cooper, H., Hedges, L. V., & Valentine, J. C. (2019). The handbook of research synthesis and meta-analysis. *Russell Sage Foundation*.

Duval, S., & Tweedie, R. (2000). Trim and fill: a simple funnel‐plot–based method of testing and adjusting for publication bias in meta‐analysis. *Biometrics*, *56*(2), 455-463. <https://doi.org/10.1111/j.0006-341X.2000.00455.x>

Egger, M., Smith, G. D., Schneider, M., & Minder, C. (1997). Bias in meta-analysis detected by a simple, graphical test. *BMJ*, *315*(7109), 629-634. <https://doi.org/10.1136/bmj.315.7109.629>

Harrer, M., Cuijpers, P., Furukawa, T. A., & Ebert, D. D. (2021). Doing meta-analysis with R: A hands-on guide. *Chapman and Hall/CRC*.

Ioannidis, J. P., & Trikalinos, T. A. (2007). An exploratory test for an excess of significant findings. *Clinical trials*, *4*(3), 245-253. <https://doi.org/10.1177/1740774507079441>

Kossmeier, M., Tran, U. S., & Voracek, M. (2020). Power-enhanced funnel plots for meta-analysis: The sunset funnel plot. *Zeitschrift für Psychologie*, 228(1), 43. <https://doi.org/10.1027/2151-2604/a000392>

Lau, J., Ioannidis, J. P., Terrin, N., Schmid, C. H., & Olkin, I. (2006). The case of the misleading funnel plot. *BMJ*, *333*(7568), 597-600. <https://doi.org/10.1136/bmj.333.7568.597>

Marks-Anglin, A., & Chen, Y. (2020). A historical review of publication bias. *Research synthesis methods*, *11*(6), 725-742. <https://doi.org/10.1002/jrsm.1452>

Pietschnig, J., Penke, L., Wicherts, J. M., Zeiler, M., & Voracek, M. (2015). Meta-analysis of associations between human brain volume and intelligence differences: How strong are they and what do they mean? *Neuroscience & Biobehavioral Reviews*, *57*, 411-432. <https://doi.org/10.1016/j.neubiorev.2015.09.017>

Schwarzer, G., & Rücker, G. (2010). Statistische Methoden zur Detektion und Adjustierung von Publikationsbias. *Zeitschrift für Evidenz, Fortbildung und Qualität im Gesundheitswesen*, 104(4), 306-313. <https://doi.org/10.1016/j.zefq.2010.03.016>

Simonsohn, U., Nelson, L. D., & Simmons, J. P. (2014). p-curve and effect size: Correcting for publication bias using only significant results. *Perspectives on Psychological Science*, *9*(6), 666-681. <https://doi.org/10.1177/174569161455398>

Simonsohn, U., Simmons, J. P., & Nelson, L. D. (2015). Better P-curves: Making P-curve analysis more robust to errors, fraud, and ambitious P-hacking, a Reply to Ulrich and Miller (2015). *Journal of Experimental Psychology: General*, *144*(6), 1146–1152. <https://doi.org/10.1037/xge0000104>

Shi, L., & Lin, L. (2019). The trim-and-fill method for publication bias: practical guidelines and recommendations based on a large database of meta-analyses. *Medicine (Baltimore)*, *98*(23), e15987. <https://doi.org/10.1097/md.0000000000015987>

Siegel, M., Eder, J. S. N., Wicherts, J. M., & Pietschnig, J. (2022). Times are changing, bias isn’t: A meta-meta-analysis on publication bias detection practices, prevalence rates, and predictors in industrial/organizational psychology. *Journal of Applied Psychology, 107*(11), 2013-2039*.* <https://doi.org/10.1037/apl0000991>

Stanley, T. D., & Doucouliagos, H. (2014). Meta‐regression approximations to reduce publication selection bias. *Research synthesis methods*, *5*(1), 60-78. <https://doi.org/10.1002/jrsm.1095>

van Aert, R. C., & van Assen, M. A. (in press). Correcting for publication bias in a meta-analysis with the *p*-uniform* method. <https://doi.org/10.31222/osf.io/zqjr9>

van Assen, M. A., van Aert, R., & Wicherts, J. M. (2015). Meta-analysis using effect size distributions of only statistically significant studies. *Psychological methods*, *20*(3), 293. <https://doi.org/10.1037/met0000025>

Vevea, J. L., & Woods, C. M. (2005). Publication bias in research synthesis: sensitivity analysis using a priori weight functions. *Psychological methods*, *10*(4), 428. <https://doi.org/10.1037/1082-989X.10.4.428>

**S4. Manual for the Newcastle-Ottawa Scale adapted for cross-sectional studies**

Herzog, R., Alvarez-Pasquin, M., Diaz, C., Del Barrio, J., Estrada, J., & Gil, A. (2013). Newcastle-Ottawa Scale adapted for cross-sectional studies. *BMC Public Health*, *13*, 154.

**Selection:** (Maximum 5 stars)

1) Representativeness of the sample:

a) Truly representative of the average in the target population. * (all subjects or random sampling)
b) Somewhat representative of the average in the target population. * (non-random sampling) c) Selected group of users.
d) No description of the sampling strategy.

2) Sample size:

a) Justified and satisfactory. *

b) Not justified.

3) Non-respondents:

a) Comparability between respondents and non-respondents characteristics is established, and the

response rate is satisfactory. *
b) The response rate is unsatisfactory, or the comparability between respondents and non-

respondents is unsatisfactory.
c) No description of the response rate or the characteristics of the responders and the non-

responders.

4) Ascertainment of the exposure (risk factor):

a) Validated measurement tool. **

b) Non-validated measurement tool, but the tool is available or described.* c) No description of the measurement tool.

**Comparability:** (Maximum 2 stars)

1) The subjects in different outcome groups are comparable, based on the study design or analysis. Confounding factors are controlled.

a) The study controls for the most important factor (select one). *

b) The study control for any additional factor. *

**Outcome:** (Maximum 3 stars)

1) Assessment of the outcome:

a) Independent blind assessment. **

b) Record linkage. **

c) Self report. *
d) No description.

2) Statistical test:

a) The statistical test used to analyze the data is clearly described and appropriate, and the

measurement of the association is presented, including confidence intervals and the probability level (p value). *

b) The statistical test is not appropriate, not described or incomplete.

**Table S4.** *Quality of included primary studies assessed with the Newcastle-Ottawa Scale.*

|  | Selection | | | | Comparability | Outcome | |  | |
| --- | --- | --- | --- | --- | --- | --- | --- | --- | --- |
| Study | 1. | 2. | 3. | 4. |  | 1. | 2. | | Total (10) |
| Bergomi et al. (2022) | 0 | * | 0 | ** | * | ** | * | | 7 |
| Coppola et al. (2015) | 0 | 0 | 0 | * | 0 | * | * | | 3 |
| D’Alessandro et al. (2017) | 0 | 0 | 0 | * | 0 | * | * | | 3 |
| Grylls et al. (2018) | 0 | 0 | 0 | ** | 0 | ** | * | | 5 |
| Paprad et al. (2020) | 0 | 0 | 0 | ** | * | ** | * | | 6 |
| Rafiee et al. (2020) | 0 | 0 | 0 | * | 0 | * | * | | 3 |
| Stillova et al. (2021) | 0 | 0 | 0 | ** | 0 | ** | * | | 5 |
| Vibrasiute (2017) | 0 | 0 | 0 | ** | * | ** | * | | 7 |
